# A Novel Artificial Neuron-Like Gas Sensor Constructed from CuS Quantum Dots/Bi_2_S_3_ Nanosheets

**DOI:** 10.1007/s40820-021-00740-1

**Published:** 2021-12-02

**Authors:** Xinwei Chen, Tao Wang, Jia Shi, Wen Lv, Yutong Han, Min Zeng, Jianhua Yang, Nantao Hu, Yanjie Su, Hao Wei, Zhihua Zhou, Zhi Yang, Yafei Zhang

**Affiliations:** Key Laboratory of Thin Film and Microfabrication (Ministry of Education), Department of Micro/Nano Electronics, School of Electronic Information and Electrical Engineering, Shanghai Jiao Tong University, Shanghai, 200240 People’s Republic of China

**Keywords:** Artificial neuron-like gas sensor, Heterostructure design, Nitrogen dioxide detection, Wearable device

## Abstract

Real-time rapid detection of toxic gases at room temperature is particularly important for public health and environmental monitoring. Gas sensors based on conventional bulk materials often suffer from their poor surface-sensitive sites, leading to a very low gas adsorption ability. Moreover, the charge transportation efficiency is usually inhibited by the low defect density of surface-sensitive area than that in the interior. In this work, a gas sensing structure model based on CuS quantum dots/Bi_2_S_3_ nanosheets (CuS QDs/Bi_2_S_3_ NSs) inspired by artificial neuron network is constructed. Simulation analysis by density functional calculation revealed that CuS QDs and Bi_2_S_3_ NSs can be used as the main adsorption sites and charge transport pathways, respectively. Thus, the high-sensitivity sensing of NO_2_ can be realized by designing the artificial neuron-like sensor. The experimental results showed that the CuS QDs with a size of about 8 nm are highly adsorbable, which can enhance the NO_2_ sensitivity due to the rich sensitive sites and quantum size effect. The Bi_2_S_3_ NSs can be used as a charge transfer network channel to achieve efficient charge collection and transmission. The neuron-like sensor that simulates biological smell shows a significantly enhanced response value (3.4), excellent responsiveness (18 s) and recovery rate (338 s), low theoretical detection limit of 78 ppb, and excellent selectivity for NO_2_. Furthermore, the developed wearable device can also realize the visual detection of NO_2_ through real-time signal changes.

**Supplementary Information:**

The online version contains supplementary material available at 10.1007/s40820-021-00740-1.

## Highlights

- An ultra-sensitive capture of NO_2_ molecules and fast charge collection and transfer has been realized by constructing the model of artificial neuron-likegas sensing structure based on CuS quantum dots (QDs)/Bi_2_S_3_ nanosheets (NSs)realizes.
- Simulation analysis revealed that CuS QDs and Bi_2_S_3_NSs can be used, respectively, as the main adsorption sites and charge transport pathways, thus leading to a greatly enhanced gas capture ability and charge conduction performance of NO_2_.

**Supplementary Information:**

The online version contains supplementary material available at 10.1007/s40820-021-00740-1.

## Graphical abstract

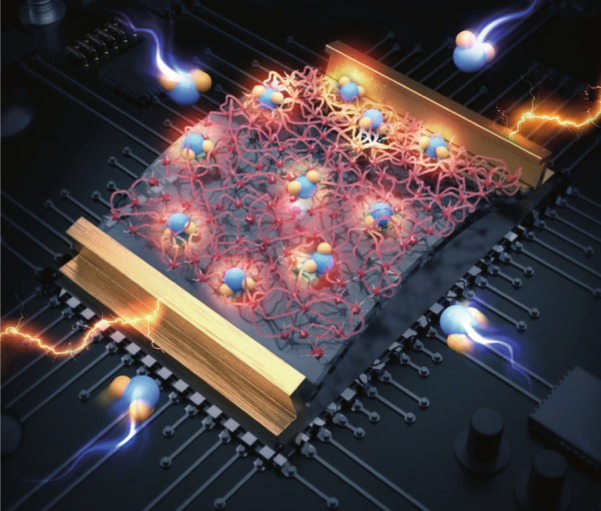

## Introduction

Over the last decades, the gradual development of industrialization and urbanization leads to the deterioration of environmental quality, especially the air condition [1, 2]. In order to cope with the timely detection of toxic and hazardous gases and to prevent the related diagnosis of respiratory injuries and diseases, the active development of low-cost, durable, high-performance gas sensors has become the focus of current research [3, 4]. With the development of the “Internet of Things” and artificial neural networks, the design of wearable gas sensors provides a promising strategy to broaden the practical applications by combining real-time monitoring and big data analysis, which allows the device applicable to the early detection of any health hazards [5–8]. Therefore, exploring wearable devices with improved room temperature detection ability ranks as the key to expand the field of gas sensing.

Similarities between biological structures and artificial neural-like networks have inspired the development of neuron-based mechanical sensing and optical sensing [9–11]. The olfactory neurons that exist in the olfactory bulb of higher animals play a key role in extracting olfactory signals through gas sensing [12]. When the human body’s olfactory receptors receive a certain intensity of stimulation, it will cause changes in the cell membrane surface potential to trigger action potentials, convert chemical signals into electrical signals, and conduct signal analysis along the nerve fibers to the central system. A large number of olfactory sensory neurons and rapid potential changes are essential for high spiritual sensitivity. Inspired by this, it is very meaningful to construct an artificial neuron-like sensor model that is different from traditional bulk sensitive materials and contains a large number of adsorption sites and fast charge transport channels.

Among active sensing materials for artificial neurons, binary or heterogeneous structures constructed from two-dimensional (2D) materials can achieve rapid response, which is of great significance for room temperature wearable sensing in practical applications. Bismuth sulfide (Bi_2_S_3_) with the direct bandgap of 1.3 eV, high carrier mobility, and excellent environment-friendly characteristics received potential attention in sensing applications [13, 14]. As a member of the stibnite family, the layered structure of Bi_2_S_3_ is projected along the *b*-axis direction by atomic-scale ribbons, which are fixed together by van der Waals (vdW) forces. Bulk Bi_2_S_3_ can be separated into two-dimensional (2D) layer structures along the (010) crystal facets maintained by weak vdW forces [15, 16]. Unfortunately, like other similar 2D gas-sensitive materials, 2D-layered Bi_2_S_3_ exhibits poor recovery properties at room temperature, which severely limits its widespread applications [17, 18]. In addition, recent studies have also proved that CuS as a p-type semiconductor material can be used alone or as a second-phase modifier to achieve rapid detection of trace gases at room temperature [19, 20]. Therefore, modifying the surface of 2D Bi_2_S_3_ nanosheets (NSs)with OD CuS quantum dots (QDs) can realize the scattered construction of sensitive points on the neuron-like network and develop a neuron-like olfactory system that is different from the traditional inefficient adsorption detection-type sensors. This will effectively help to achieve rapid and sensitive detection at room temperature.

Herein, we first report a sensor model inspired by the biological olfactory neuron system, which realizes the efficient detection of NO_2_ by uniformly loading CuS QDs on Bi_2_S_3_ NSs. Density functional theory (DFT) reveals the charge distribution and transfer process of 0D-2D heterojunction neurons before and after contacting gas molecules. As a result, the 0D p-type CuS quantum dots with higher adsorption energy for NO_2_ molecules are confirmed to be the main adsorption sites for the target gas, and the 2D n-type Bi_2_S_3_ nanosheets provide the main transfer path for the charge carriers. Subsequent experiments confirmed that appropriately high-tuned 0D QDs were loaded on the carrier as second-phase particles. Attribute to the numerous active adsorption sites and a large amount of charge transfer, interface electronic interaction can be greatly enhanced, which facilitates the action of gas molecules on the material surface [21, 22]. Moreover, the design of this n–p heterojunction can effectively increase the rate of charge transfer and has realized the ultra-sensitive response of artificial neurons to the stimulation of gas molecules. Therefore, this study uses the synergistic effect between high adsorption energy and fast charge transfer to simulate the biological olfactory detection process by rationally constructing heterojunction neurons and further realizes the sensitive monitoring of NO_2_. Finally, the sensing materials have been further integrated into our self-developed wearable gas detection equipment. The real-time display and cloud storage of detection signals on smart device applications through data collection and Bluetooth wireless transmission has been fully achieved. Our work sheds light on the rational design of bio-simulation intelligence, wearable, and wireless sensing equipment toward the “neuron-like sensitive intelligence era.”

## Experimental Section

### Synthesis of Bi_2_S_3_ NSs

30 mg of commercial bulk Bi_2_S_3_ powder was added to a 25 mL flask. 10 mL of aqueous ethanol in various volume ratios was added as a dispersion solvent. Ultrasonication was performed for 8 h. Then, the precipitate was removed by centrifugation treatment.

100 mg of Bi_2_S_3_ powder and acetonitrile was ground with agate mortar for 2 h [23]. The samples were dried with a vacuum oven and then redistribute in a ethanol aqueous solution (70 vol%, 50 mL, Fig. S1). After ultrasonic treatment at 200 W for 3 h, the supernatant was extracted by centrifugation at 1500 rpm for 20 min to collect Bi_2_S_3_ NSs [17, 24].

### Synthesis of CuS QDs/Bi_2_S_3_ NSs and CuS

0.0138 g of Cu(NO_3_)_2_ and 0.01 g of Na_2_S∙9H_2_O were dissolved in 20 mL of ethanol aqueous solution, respectively. Then, 0.1 g Bi_2_S_3_ NSs were dispersed in 10 mL ethanol and stirred continuously with an ultrasonic instrument for 30 min. The dispersion was mixed with the above two solutions, respectively, for reaction, and finally, the prepared CuS QDs/Bi_2_S_3_ NSs were collected after centrifugation and washing [22]. Following the same procedure as above, bare CuS was obtained with the addition of only Cu(NO_3_)_2_ and Na_2_S∙9H_2_O. The as-prepared products were named as BC-2.5, BC-3, BC-4, BC-5, BC-6, BC-7.5, BC-10, and BC-20 corresponding to 2.5, 3, 4, 5, 6, 7.5, 10, and 20 wt% of CuS, respectively.

### Sensor Device Fabrication and Sensing Measurements

The sensor device uses a flexible electrode sputtered with Au fingers on polyimide (PI) substrate. Clean the electrode surface alternately with deionized water and ethanol to promote the contact between the sensing membrane and the electrode. To fabricate the gas sensor, the ethanol dispersion of CuS QDs/Bi_2_S_3_ NSs with a concentration of 70 vol% was sprayed onto the PI electrode using inkjet printing equipment. Finally, the as-prepared sensors were dried in the vacuum oven.

The details of the gas-sensitive test system can be found in our previous paper [25]. In simple terms, the chamber was purged with dry compressed air before the measurement to stabilize the baseline signal. Subsequently, the mass flow controller (MFC) was used to control the flow of NO_2_ and dry compressed air with an initial concentration to obtain NO_2_ dilution gas of different concentrations with a flow rate of 1 standard liter/min (SLM). The Agilent 4156C analyzer was utilized to detect current changes of gas sensors at room temperature (25 °C). The response value is defined as *R*_a_/*R*_g_, which are resistances of the sensor when exposed to compressed air and NO_2_, respectively. In addition, the response and recovery time (*τ*_res_ and *τ*_rec_) are the time required for the sensor to fully respond and recover to 90% after being exposed to NO_2_.

### Characterizations

Crystallinity and morphology of as-prepared CuS QDs/Bi_2_S_3_ NSs sample were analyzed with the X-ray diffractometry (XRD, D8 ADVANCE, Bruker), transmission electron microscopy (TEM, JEM-2100, JEOL, Japan), and scanning electron microscopy (SEM, Carl Zeiss Ultra Plus, Germany). The chemical components and band structures were studied by X-ray photoelectron spectroscopy and ultraviolet photoelectron spectroscopy (XPS and UPS, Kratos Axis Ultra^DLD^). Ultraviolet–visible (UV–vis) absorption spectra (Perkin-Elmer, USA) were utilized to characterize bandgaps of materials. The Raman spectra were acquired on the confocal Raman microscope (RENISHAW, England). Mott–Schottky plots measurements were performed with an electrochemical workstation (CHI 760E, Shanghai Chenhua).

## Results and Discussion

### DFT Calculation of Neuron-like Sensor Network Inspired by Olfactory Sensory Neurons

**Fig. 1:**
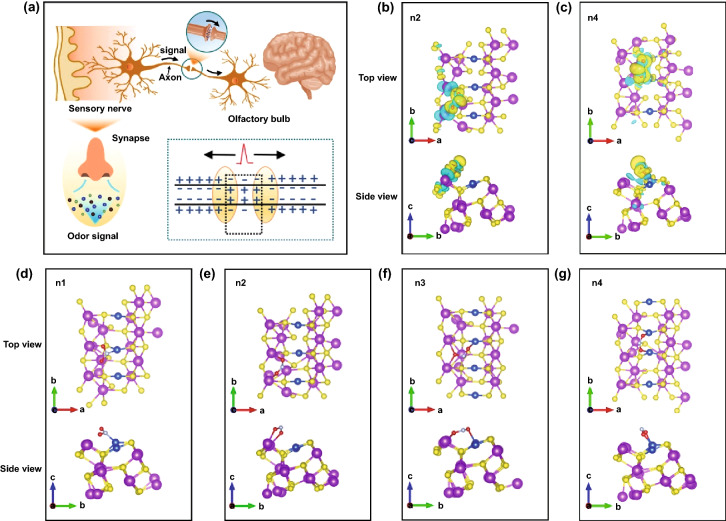
**a** Schematic diagram of charge change of biological olfactory system during stimulation. Sensing mechanism and the possible interaction energy of gases with configurations on the CuS QDs/Bi_2_S_3_ NSs using DFT calculations. **b** and **c** Diagrams of charge density difference of NO_2_ on CuS-Bi_2_S_3_ with n2 and n4 binding structure for side and top view, where the charge density isosurfaces of blue and yellow are 0.0015 and -0.0015 e Å^−3^, respectively. **d-g** Binding structures of NO_2_ on CuS-Bi_2_S_3_

It is of great significance to deeply understand the perception process of the olfactory organs of organisms to odor molecules and to compare the mechanism of artificial neuron gas sensors with it. As shown in Fig. 1a, when the human body’s olfactory receptors receive a certain intensity of stimulation, it will cause changes in the surface potential of the cell membrane to trigger action potentials, convert chemical signals into electrical signals, and conduct analysis along nerve fibers to the central system and generate corresponding reaction [12]. A large number of sensory neurons in the biological olfactory system can realize super-sensitivity to gas molecules, and the timely conduction of electric potentials realizes the rapid transmission of signals. Inspired by this, the CuS QDs/Bi_2_S_3_ NSs-based neuron-like sensing model constructed by loading the 0D adsorption sites on the 2D semiconductor transmission channel has great potential to realize highly sensitive sensing of NO_2_ molecules.

In order to reveal the role of artificial olfactory neuron sensing interface in the process of NO_2_ sensing, DFT was used to analyze the adsorption and charge transfer processes of NO_2_. Four binding structures of NO_2_ on CuS-Bi_2_S_3_ are studied in our work, and the adsorption conformation is shown in Fig. 1d–g. The corresponding binding energies (${E}_{b}= {E}_{N{O}_{2}}+ {E}_{CuO-{Bi}_{2}{S}_{3} }- {E}_{total}$) are 1.15, 1.70, 1.45, and 2.25 eV. The results showed that NO_2_ prefers to bind to CuS-Bi_2_S_3_ with forming N-Cu bonds, and the type of Bi-O binding structure has the smallest binding energy, and the length of M(Bi/Cu)-O bond is listed in Table S1. To explain the difference of binding property, charge density difference, Bader charge transfer, and M–O bond length are analyzed. Clearly, the binding structure with Cu–O bond structure is favorable with the highest binding. As displayed in Fig. 1b, c and Table S2, for Bi-O structure, the charge is mainly transferred between Bi and O atoms, while for Cu–O, it is between Cu and O atoms. Bader charge analysis shows that CuO and NO_2_ have greater charge transfer, which may be beneficial to the binding of NO_2_, which is consistent with the relationship between adsorption energy. This series of DFT results proved the conclusion that 0D CuS QDs are used as NO_2_ sensitive points, and 2D Bi_2_S_3_ NSs are used as a charge transport network to achieve an efficient gas sensing response. Therefore, in the sensing process of artificial olfactory neurons, the CuS QDs/Bi_2_S_3_ NSs heterostructure sensing unit can efficiently adsorb gas molecules and quickly transfer charge signals, which undoubtedly greatly improves the efficient sensing of NO_2_.

### CuS QDs/Bi_2_S_3_ NSs-Based Sensor Inspired by Biological Olfactory Neurons

**Fig. 2:**
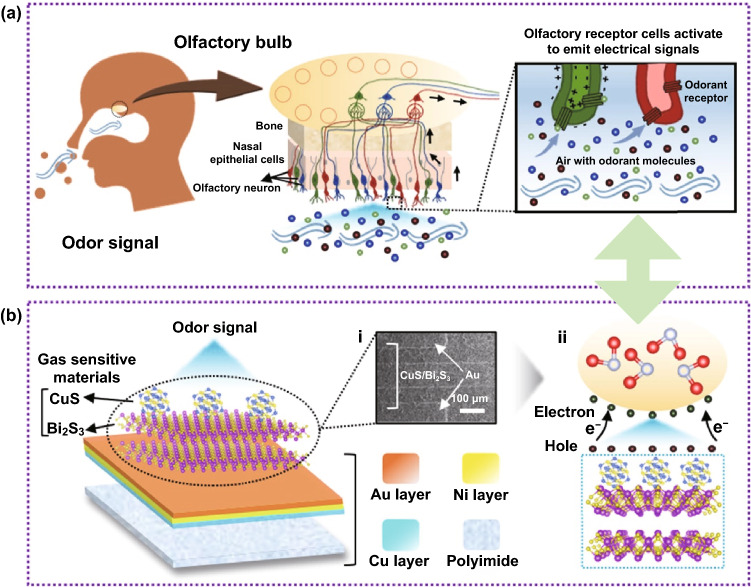
**a** Schematic diagram of biological olfactory system. **b** Schematic diagram of artificial olfactory neuron sensing based on CuS QDs/Bi_2_S_3_ NSs heterostructure

Figure 2a shows the process of recognition and perception of the smell of the external environment by organisms. When the receptors located in the olfactory bulb of the human body are stimulated by a certain intensity of gas molecules, olfactory receptor cells release electrical signals. The electrical signal is then conducted in the olfactory glomerulus and finally sent to the brain area for perception and recognition [12].

Inspired by the sensory nervous system, we tried to prove that the sensory synapses of the artificial neuron structure have the same perceptual behavior as biological perception. The schematic diagram of olfactory artificial neuron is revealed in Fig. 2b. It includes an ultra-thin PI substrate deposited with interdigital electrodes and a sensitive detection unit based on CuS QDs/Bi_2_S_3_ NSs heterostructure. In the gas sensor, 0D-2D CuS QDs/Bi_2_S_3_ NSs are stacked on an ultra-thin PI substrate deposited with Au electrodes by inkjet printing (optical image in Fig. 2b–i). Besides, NO_2_ molecules with strong electron deprivation properties are adsorbed on the surface of artificial neurons in the sensing process, which triggers changes in the surface charge distribution by depriving them of charges and finally leads to the generation of electrical signals (Fig. 2b-ii).

### Artificial Neuron-like Sensing Layer Based on CuS QDs/Bi_2_S_3_ NSs Heterostructure

**Fig. 3:**
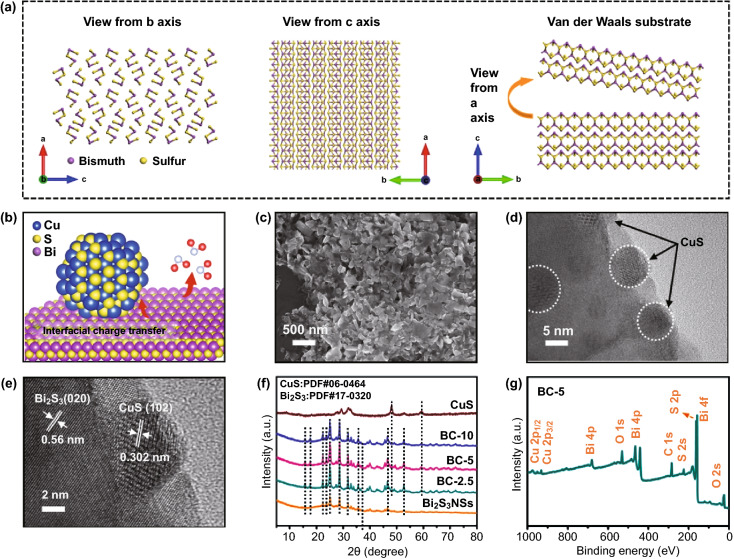
CuS QDs/Bi_2_S_3_ NSs heterostructure and characterization of CuS and Bi_2_S_3_ NSs. **a** Schematic diagram of crystal structure and fracture-exfoliation process of Bi_2_S_3_ bulk material with selective orientation. **b** Schematic representation of the atomic structure of CuS QDs/ Bi_2_S_3_ NSs. **c-e** SEM, TEM and HR-TEM images of BC-5. **f** XRD patterns of CuS QDs/Bi_2_S_3_ NSs, CuS and Bi_2_S_3_ NSs. **g** Full XPS survey spectrum of BC-5

To achieve ultra-sensitive detection, it is particularly important to construct a reasonable sensor layer of artificial olfactory neurons. As schematically shown in Fig. 3a, in brief, ultrasonic stripping of bulk Bi_2_S_3_ was executed in a mixed solution of anhydrous ethanol and deionized water for the first time without any further complicated cleaning treatment. In stark contrast to commercial bulk Bi_2_S_3_ (Fig. S2), the lateral size and thickness of Bi_2_S_3_ NSs were reduced from a few micrometers to the nanometer scale upon grinding and ultrasonic treatment.

Subsequently, a simple two-step chemical precipitation technique was designed to hybridize CuS QDs to the surface of Bi_2_S_3_ NSs, and its chemical structure is shown in Fig. 3b. The composite structures of BC-5 (a series of CuS QDs modified Bi_2_S_3_ NSs hybrid materials with different CuS content are numbered BC-n, where n is the mass percentage of CuS QDs in the hybrid material, *n* = 2.5, 3, 4, 5, 6, 7.5, 10, and 20) were studied by SEM and TEM. As shown in Fig. 3c, the morphology of BC-5 keeps the same as the bare Bi_2_S_3_ NSs, which still exhibits a nanosheet appearance as the lateral size of ca. 200 nm without an observable change in morphology upon the loading of CuS. The thin 2D structure supplies a boosted surface area for the CuS QDs loading.

CuS QDs (≈ 8 nm) uniformly distributed on the surface of Bi_2_S_3_ NSs are observed in the TEM image (Fig. 3d). Attribute to the higher surface energy at the edge positions of nanosheets and the individual distribution of quantum dots without agglomerates or clusters, the CuS QDs are prone to be attached to the edge positions of Bi_2_S_3_ NSs. The intimate attachment of CuS QDs on Bi_2_S_3_ NSs demonstrates the strong bonding and interacting heterogeneous structures at the interface, which is essential for their enhanced sensing performance [22, 26].

Based on the high-resolution TEM (HR-TEM), a 0.56 nm lattice spacing is found for Bi_2_S_3_ NSs and BC-5 (Figs. S2d and 3e), which corresponds to the (020) facets of Bi_2_S_3_ crystal and again supports the process of selective orientation stripping of Bi_2_S_3_ NSs [13]. Additionally, a lattice fringe spacing of 0.304 nm is also found in Fig. 3e, which can be ascribed to the crystallographic facet (102) of CuS. The confirmation of these lattices further proves that the construction of CuS QDs and Bi_2_S_3_ NSs forms a heterostructure rather than a simple physical mixing [27]. In addition, the presence of Bi_2_S_3_ NSs results in a more uniform loading of CuS QDs on its surface. The detailed microstructure of the irregular CuS without substrate nanosheets is indicated in Fig. S4c. The XRD confirmed the structural regularity of bulk material and the crystal planes of orthorhombic Bi_2_S_3_. Exfoliated Bi_2_S_3_ NSs show preferential orientation along the (010) facet, which increases the intensity of the peaks including (130), (310) and (020) (Fig. S3a). In addition, a decrease in the overall peak intensity of the product sample has been observed, demonstrating that the exfoliation process distorts the crystal lattice [16, 28]. The similar Raman spectrum patterns of bulk material and thin layer are displayed in Fig. S3b, Supporting Information. The transverse in-plane A_g_ vibration peaks occurring at 188 and 240 cm^−1^ and the longitudinal B_1g_ vibrations at 168 and 265 cm^−1^ coincide with the values reported previously [29]. It is noteworthy that Bi_2_S_3_ NSs have a similar Raman signal as bulk material, further confirming that the properties of nanosheets remain unchanged after liquid-phase exfoliation process.

XRD pattern of the CuS QDs/Bi_2_S_3_ NSs (Fig. 2f) is specifically indexed into orthorhombic Bi_2_S_3_ and hexagonal CuS [27, 30]. As a blank control, bare CuS was prepared without the loading of Bi_2_S_3_ NSs. The bare CuS has revealed a single-phase structure in XRD and Raman tests as expected in the pure hexagonal phase (Fig. S4) [31]. X-ray photoelectron spectroscopy (XPS) measurements have been further used to study the elements in CuS QDs/Bi_2_S_3_ NSs and other products. As displayed in Figs. 3g and S5, the full XPS spectrum of the heterostructure includes the corresponding peaks that are associated with Bi_2_S_3_, while the peak at 931.9 and 951.7 eV, respectively, supports the presence of Cu 2p_3/2_ and 2p_1/2_ in CuS [32]. This confirms the successful formation of CuS and Bi_2_S_3_ in the composite product, which shows no difference with XRD results.

**Fig. 4:**
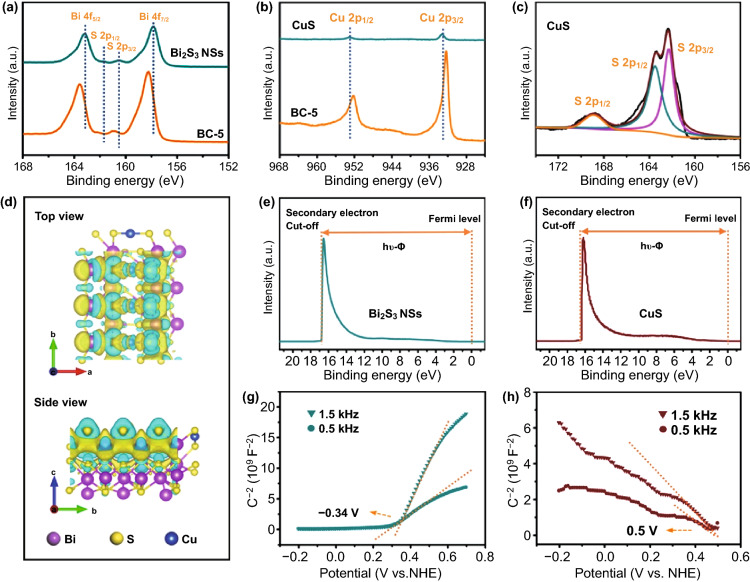
Analysis of interface charge state of the heterostructure. **a-c** High-resolution XPS spectra of Bi 4f, Cu 2p and S 2p of the BC-5, CuS, and Bi_2_S_3_ NSs. **d** Charge distribution at the interface of CuS QDs and Bi_2_S_3_ NSs calculated by density functional theory. UPS spectra of **e** Bi_2_S_3_ and **f** CuS. Mott − Schottky plots for **g** Bi_2_S_3_ NSs and **h** CuS

To consider the potential distribution of the resting state of the artificial neuron sensor layer before being stimulated by the olfactory sense, the interface interaction between the CuS QDs and Bi_2_S_3_ NSs was further studied. The electronic structures of the composite products and the pure phase monomer constituent elements were studied by high-resolution XPS measurement. As displayed in Fig. 4a, two separated peaks in the Bi 4f XPS spectrum at 158.3 and 163.6 eV are 4f_7/2_ and 4f_5/2_ hybridized orbitals of the Bi 4f orbital energy level for BC-5 sample, respectively [33]. Compared with bare Bi_2_S_3_ NSs, the peak position shifts to the high binding energy, indicating that the electron density on the Bi_2_S_3_ surface in BC-5 nanocomposites is reduced. Two peaks at 932.1 and 952.2 eV of Cu 2p (Fig. 4b) can be regarded as the Cu^2+^ [30]. Compared to bare CuS, the peak position shifts about 1 eV toward the lower binding energy, indicating an elevated electron density on the Cu surface in the BC-5 nanocomposite. Meanwhile, the S 2p XPS spectra in Fig. 4a, c show similar results. The results of these peak shifts prove the formation of heterogeneous structures. The charge transfer between the interfaces is from Bi_2_S_3_ to CuS. Moreover, the chemical bonds formed between CuS QDs and Bi_2_S_3_ NSs lead to the rapid migration of interfacial charges and induce the appearance of strong polarization and strong electric fields, which help to enhance the adsorption and desorption efficiency of NO_2_ [21, 26, 34].

To understand the charging behavior of the CuS QDs/Bi_2_S_3_ NSs, the charge density difference at the interface was studied using DFT calculation. As exhibited in Fig. S6 in Supporting Information, three Cu atoms in the CuS QDs/Bi_2_S_3_ NSs composite structure are bonded at the (010) crystal plane S–S bridge site. From the three-dimensional migration diagram of the CuS QDs/Bi_2_S_3_ NSs interfacial charge in Fig. 4d, the CuS QDs/Bi_2_S_3_ NSs interfacial charge shows a strong polarization, efficient electron–hole separation, and redistribution state. The differential charge density and bader charge transfer values are displayed in Table S3, showing a strong bonding formed between CuS and Bi_2_S_3_, and the bader charge transfer value is 3.371 e, which illustrates the correctness of this structural model and proves that the interfacial charge is transferred from Bi_2_S_3_ to CuS.

Besides, to gain a deeper understanding of the redistribution of interfacial charges and to reveal the transition process of Bi_2_S_3_ NSs upon CuS QDs loaded on the surface, the energy band structure of the interface materials was further characterized. Herein, ultraviolet photoelectron spectroscopy (UPS) measurements are employed to establish the specific energy band location of heterojunction, where the position of the conduction band (CB), the Fermi energy level (E_f_), and the valence band maximum (VB) are determined by E_f_ = 21.22 eV − E_cutoff_ (E_cutoff_ is the energy at which the secondary light emission starts), E_V_ = 21.22 eV − [E_cutoff_ − (E_f_ − E_VM_)] (E_VM_ is the valence band maximum). Then, the bandgap values of Bi_2_S_3_ and CuS were, respectively, determined to be 1.3 and 1.8 eV from the UV–Vis diffuse reflectance spectra and the corresponding Tauc plots (Fig. S8) [35]. In addition, the Mott–Schottky technique is employed to study the semiconductor properties of interfacial materials, as well as flat-band potentials. As shown in Fig. 4g, h, the positive and negative slope indicates the n-type and p-type behavior of Bi_2_S_3_ and CuS, while its flat-band potential is calculated as -0.34 V, as well as 0.5 V, respectively [34, 36].

Moreover, the specific energy band locations of heterojunction materials are described in Fig. S9 with a corresponding model to explain the interfacial charge redistribution. When the two different semiconductors at the interface come into contact, due to the distance between the E_f_ of Bi_2_S_3_ and the vacuum electron energy level (E_vac_) of -4.37 eV, driven by the thermodynamic difference between the work function of Bi_2_S_3_ (-4.37 eV) and CuS (-4.79 eV), electrons will spontaneously transfer from Bi_2_S_3_ to CuS across the interface, thus forming an electron depletion layer on the surface of Bi_2_S_3_. On the contrary, holes of p-type CuS near the interface tend to diffuse into n-type Bi_2_S_3_, thus establishing a hole depletion layer on the CuS surface. The electron–hole diffusion continues until the heterojunction establishes a uniform E_f_, which conduces to the energy band bending at the interface. Furthermore, the CuS QDs/Bi_2_S_3_ NSs p–n junction exhibits a type-II band structure, which is conducive to inhibit electron–hole recombination [37].

### Sensing Performance of the Artificial Olfactory Neuron-like Sensor

**Fig. 5:**
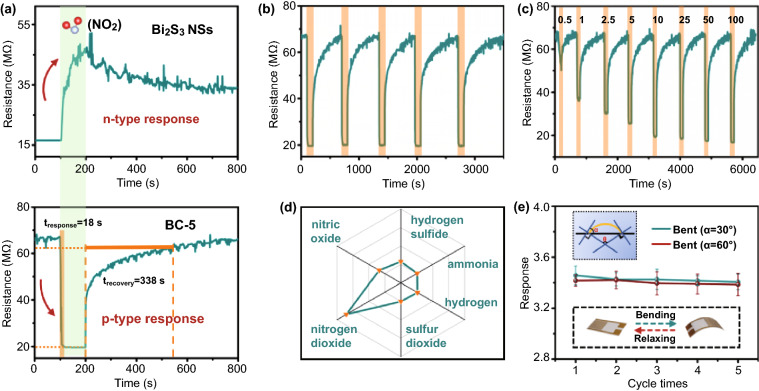
NO_2_ sensing performances BC-5-based sensor. **a** Resistance variation for the device to NO_2_ at 10 ppm concentration. **b** Response-recovery curve of the device for 5 cycles to 10 ppm NO_2_. **c** Transient response-recovery curve for the device to NO_2_ with different concentrations. **d** Selectivity for the device to 10 ppm different target gases. **e** Response graph and response error bar for the device to 10 ppm NO_2_ with different bending angles

To assess the effect of interfacial electrons on materials after the construction of heterogeneous structures, the sensing material was sprayed on the electrode surface using inkjet printing technology, and the change of its gas-sensitive performance was studied in a homemade dynamic sensing system (Figs. S10 and S11). The variation in sensing performance of starting bulk materials, Bi_2_S_3_ NSs, and CuS QDs/Bi_2_S_3_ NSs with different weight ratios for 10 ppm NO_2_ is displayed in Figs. 5a, S12 and S13. The exfoliated Bi_2_S_3_ NSs showed greater electrical resistance compared to the bulk material, which was ascribed to the large number of defects introduced after liquid-phase exfoliation (Table S4) [17, 26]. The pristine Bi_2_S_3_ displayed an increase in resistance immediately after being exposed to NO_2_, indicating an n-type semiconducting property as identical to the Mott–Schottky (M–S) test results. Meanwhile, the larger specific surface area after exfoliation enriches the number of the active sites on the samples, resulting in increased response and reduced recovery performance.

When CuS QDs are loaded at the lower concentrations (2.5 wt% and 3 wt%), an increase in the sample resistance, as well as a decrease in the response magnification, can be observed, which can be attributed to the recombination of electrons in Bi_2_S_3_ NSs with holes from the p-type CuS. As the loading amount of CuS QDs further increases (beyond 4 wt%), the sensor exhibits a decreasing resistance change in the presence of NO_2_, indicating that the n-type Bi_2_S_3_ has been completely transformed into a p-type complex by the multiple (hole) injection of p-type CuS. This result suggests that the CuS QDs/Bi_2_S_3_ NSs possess a complete transition of n- to p-type in semiconductor properties. When the CuS QDs loading on the surface of Bi_2_S_3_ NSs is further increased (over 6 wt%, until it reaches bare CuS), a further decrease in the surface resistance of sensitive materials is observed. This phenomenon is attributed to the further increase in the majority carrier (hole) concentration of the p-type semiconductor composite product. The continuous improvement of the conductivity of sensitive materials makes the resistance change more difficult to be observed, which is manifested as a decrease in the response rate [38].

By comparing the response and recovery properties of the heterojunction-based sensors with different CuS QDs loadings to 10 ppm NO_2_, BC-5 has been identified as the optimal device with a response amplification of 3.4 and ultra-fast response and recovery rates (18 and 338 s, respectively) without the assistance of any additional means. Our BC-5 has shown a grand advance in sensing performance over the previously reported works on NO_2_ room temperature flexible sensors (Table S5). According to the previous DFT calculations, compared to the initial bulk Bi_2_S_3_ material, the reason for the superior performance of the BC-5-based sensor attributes to the construction of many sensitive points and the rapid conduction of charges.

To reveal the importance of reversibility for BC-5-based gas sensors, the cyclic test is executed to 10 ppm NO_2_. The sensing performance was kept nearly unchanged after five cycle uses (Fig. 5b). In addition, Fig. 5c shows the response-recovery curves of the BC-5-based sensor exposed to NO_2_ at concentrations of 500 ppb to 100 ppm. With the response change and error bar data summarized in Fig. S13, BC-5-based sensors exhibit excellent sensitivity and recovery capability. The theoretical limit of detection (LOD) of the device toward NO_2_ was calculated as approximately 78 ppb by the least squares method using 3RMS_noise_ divided by the slope of the low-concentration partial fit curve (Fig. S14b) [8, 39]. The fitted curve shows a dichotomous distribution due to the scattering effect of NO_2_ molecules resulting in reduced carrier mobility [40].

To study the sensing performance of the device in the real environment, we have further investigated its long-term stability and selectivity. The long-term stability of BC-5-based sensor was estimated upon exposure to 10 ppm NO_2_ for 2 months at an interval of 1 week (Fig. S14c). The response value of the device remained virtually unchanged for two months, indicating excellent long-term stability. This result indicates that the depletion layer formed at the interface by our heterostructure building process can act as a passivation layer, preventing environmental oxidation of sulfides and leading to reliable long-term stability [41, 42]. Furthermore, the selectivity of BC-5-based sensor for different gases was tested. Different analytes were investigated including 10 ppm NO_2_, H_2_S, NH_3_, SO_2_, H_2_, and NO. As shown in Figs. 5d and S15, the response value for NO_2_ was significantly higher than other test gases, indicating that the device exhibited excellent selectivity. Besides, considering the influence of humidity, the sensor was exposed to 10 ppm NO_2_ with relative humidity ranging from 0 to 80% (Fig. S16). The response value decreases from 3.4 to 1.6 as the relative humidity increased, indicating that the sensor can still work normally under high humidity conditions. The negligible decrease in response is probably arising from the fact that some H_2_O molecules occupy the sensing sites [43].

In addition, the illustration of Fig. 5e depicts a schematic diagram of the mechanical strain bending direction of the flexible device. The flexible sensor was bent at 30º and 60º followed by sensing tests in 10 ppm NO_2_ for five cycles. The response results of the device clearly showed that the bending of the flexible electrode will not reduce the property of the as-prepared device.

### Gas sensing Mechanisms of the Artificial Neuron-like Olfactory System

Based on the DFT calculations and the corresponding experimental results, we conducted an in-depth understanding and discussion on the mechanism of the artificial neuron-like sensor based on CuS QDs/Bi_2_S_3_ NSs to enhance the NO_2_ sensing performance.

First of all, the DFT calculation results prove that in the neuron-like sensor model constructed by the olfactory sensory neurons, a large number of dispersed OD CuS QDs can be used as NO_2_ sensitive points to efficiently capture gas molecules. The 2D Bi_2_S_3_ NSs as a conductive network and a heterojunction substrate can achieve rapid charge transfer through synergy.

Secondly, the artificial olfactory neuron sensor based on the heterogeneous structure of CuS QDs/Bi_2_S_3_ NSs has the same charge transfer method as the biological olfactory perception process in the resting state and after being stimulated by gas molecules. The charge distribution process of the CuS QDS/Bi_2_S_3_ NSs heterostructure itself in the resting state was studied in the previous chapter. In brief, upon intimate contact, electrons move spontaneously from Bi_2_S_3_ to CuS across the interface for the equilibrium of Fermi level, leading to the formation of the electron and hole depletion layer on the surface of Bi_2_S_3_ and CuS, respectively. In addition, the formation of a built-in electric field and the bending of energy band also occur.

As shown in Fig. S17, when exposed to NO_2_ (electron acceptor), gas molecules are adsorbed onto the CuS QDs surface and trap electrons, breaking the equilibrium of the built-in electric field formed previously. The additional holes return to CuS that change the width of the potential barrier until formed a new equilibrium. The presence of a potential barrier in the heterojunction at the interface can reflect small fluctuations in carrier concentration due to the adsorption of NO_2_ molecules as large changes in resistance, which undoubtedly facilitates the response process [44]. In addition, the built-in electric field can also accelerate the charge carrier transfer efficiency, so as to improve the sensing performance [45, 46].

Furthermore, the switch from n-type to p-type character and the construction of surface heterostructures also affects the selectivity of the device. Compared with other gases, the adsorption of NO_2_ with a higher charge density can significantly increase the heterojunction barrier and improve its sensing performance [37]. The design of this series of interfacial heterostructures and charge modulation successfully enhanced the room temperature sensing performance of the artificial olfactory neuron-like sensors for NO_2_, making it significantly better than the previously reported Bi_2_S_3_-based sensors and providing a new idea for the design of the heterogeneous interface of the biological simulation artificial olfactory neuron sensor.

### Wireless Wearable Devices and Real-time Monitoring of NO_2_

**Fig. 6:**
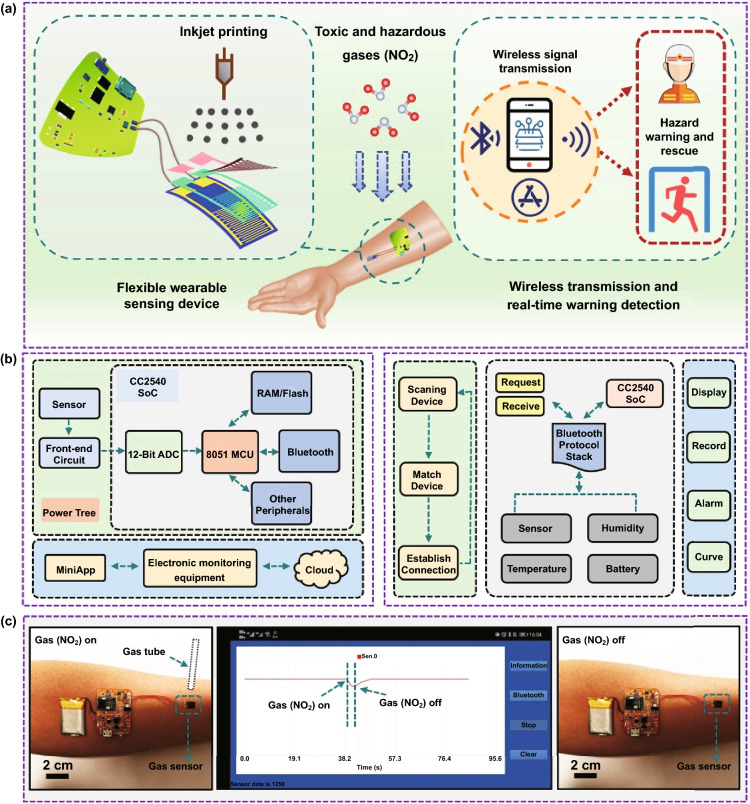
Application process of the wireless wearable sensor system. **a** Schematic diagram of structure and application concept of wireless wearable devices. **b** Circuit hardware and software logic block diagram. **c** Photographs of the change in response to NO_2_ gas by smartphone application during the actual test

In the previous content, we correlated the process of biological olfactory perception with the electrical signal generation and transmission process of the artificial neuron-like sensor in the process of detecting gas. The result of the transmission of sensory signals in the organism is the reception, analysis and response of the central nervous system of the brain. This series of processes is consistent with the sensor’s back-end information collection and real-time data extraction. Previously, many flexible gas sensors have been developed while their real-world applications in wireless and wearable electronics still confront a challenge and are rarely demonstrated [47]. Intending to design wearable applications in real time, we integrated CuS QDs/Bi_2_S_3_ NSs-based artificial neuron-like sensor, circuit, and data acquisition to develop a portable NO_2_-monitoring system (Fig. 6a).

The hardware and software block diagram of the as-prepared device system is illustrated in Fig. 6b. During the operation of the device, Bluetooth communication is first turned on, and the mobile device equipped with the self-developed Android-based application scans the surrounding Bluetooth devices and verifies and connects after finding a matching sensing device. After the connection, the application will request the data acquisition circuitry for information such as the sensor’s output voltage and real-time time. Once the data are obtained, the application can calibrate the ambient temperature and humidity, connect to a database to calculate the corresponding environmental pollution conditions, and display the relevant gas concentrations in real-time according to the requirements written. Moreover, by presetting the threshold value, the user can be warned in time when the pollution is aggravated and the gas concentration exceeds the standard.

For the working tests in this paper, a 200 mAh rechargeable lithium-polymer battery (TF402030) was used to provide the appropriate voltage for all circuits, and the actual voltage on the flexible sensing device was 3.3 V (device circuit schematic is displayed in Fig. S18). The double-sided copper-clad flexible PCB is connected to the PI flexible electrode deposited with the gold finger by flexible silver wires (Fig. S19a), ensuring the flexibility of the wearable device and the accuracy of data transmission. The entire wireless wearable sensing device was tested for NO_2_ atmosphere in a fume hood at room temperature. In the actual test procedure, the signal connection and transmission from the Bluetooth device were first performed (Fig. S19b and Video S1), followed by a drop in the resistance value on the mobile application with the tube placed beside the device, i.e., the NO_2_ signal was detected. As the gas tube is removed, the curve trajectory spontaneously returns to the baseline (Fig. 6c). Also, given the fact that the interface-modulated CuS QDs/Bi_2_S_3_ NSs were able to return the signal to baseline without external assistance, we tested a continuous response for 4 min and revealed it on a phone application to demonstrate the reproducibility (Fig. S19c and Video S2).

The results presented herein efficiently cross-fertilize the fields of biological neuron-like sensing unit design, mechanical circuit fabrication, and software development to successfully develop wearable sensing devices that are lightweight, visual, and multifunctional. The advantages of biomimetic association, cheap, and low power consumption allow for sustainable utilization, and the development of wireless data transmission coincides with the expansion of the modern Internet of Things.

## Conclusions

In summary, a self-designed bio-simulated neuron-like gas sensor that can drive wireless wearable devices for real-time monitoring of NO_2_ has been reported. First, 0D p-type CuS QDs were successfully loaded onto the surface of 2D n-type Bi_2_S_3_ NSs. Characterization analysis and DFT calculations were used for the first time to reveal that the charge transfer process of artificial neuron-like sensors in the resting state and when stimulated by gas is consistent with the process of biological olfactory perception. Moreover, the heterogeneous integration of artificial neuron-like sensor was unveiled that synergistically combines CuS QDs sites with high adsorption energy for target gas molecules and Bi_2_S_3_ NSs as fast charge transport channels. Compared with traditional bulk sensing materials, the problem of high-efficiency detection at room temperature can be effectively solved by this type of artificial neuron-like sensor through many scattered sensitive points. This exhibits an ultra-fast response (18 s) and recovery (338 s), an exceptional theoretical detection limit (78 ppb), a tunable sensing mechanism, and excellent selectivity. Second, through the low-concentration NO_2_ ventilation analysis of the wearable gas sensing device on the arm of the human subject, the visualization and real-time observation of the target gas on the smartphone app is realized. This artificial neuron-like device establishes a promising platform for the next generation of simulating complex biological nervous systems and promoting the development of artificial intelligence.

## Supplementary Information

Below is the link to the electronic supplementary material.

Supplementary file1 (DOCX 3292 kb)

Supplementary file2 (MP4 14338 kb)

Supplementary file3 (MP4 6428 kb)

## References

[1] Figueres C, Christiana PJ, Landrigan R. Fuller (2018). Tackling air pollution, climate change, and NCDs: time to pull together. Lancet..

[2] Han X, Godfrey HGW, Briggs L, Davies AJ, Cheng Y (2018). Reversible adsorption of nitrogen dioxide within a robust porous metal–organic framework. Nat. Mater..

[3] Meng Z, Stolz RM, Mendecki L, Mirica KA (2019). Electrically-transduced chemical sensors based on two-dimensional nanomaterials. Chem. Rev..

[4] Agrawal AV, Kumar N, Kumar M (2021). Strategy and future prospects to develop room-temperature-recoverable NO_2_ gas sensor based on two-dimensional molybdenum disulfide. Nano-Micro Lett..

[5] Pang Y, Yang Z, Yang Y, Ren TL (2019). Wearable electronics based on 2D materials for human physiological information detection. Small.

[6] Mamun MAA, Yuce MR (2020). Recent progress in nanomaterial enabled chemical sensors for wearable environmental monitoring applications. Adv. Funct. Mater..

[7] Tang N, Zhou C, Xu L, Jiang Y, Qu H (2019). A fully integrated wireless flexible ammonia sensor fabricated by soft nano-lithography. ACS Sens..

[8] Guo S, Yang D, Zhang S, Dong Q, Li B (2019). Development of a cloud-based epidermal MoSe_2_ device for hazardous gas sensing. Adv. Funct. Mater..

[9] Keene ST, Lubrano C, Kazemzadeh S, Melianas A, Tuchman Y (2020). A biohybrid synapse with neurotransmitter-mediated plasticity. Nat. Mater..

[10] J.R. Yu, X.X. Yang, G.Y. Gao, Y. Xiong, Y.F. Wang et al., Bioinspired mechano-photonic artificial synapse based on graphene/MoS_2_ heterostructure. Sci. Adv. **7**, eabd9117 (2021). https://advances.sciencemag.org/content/7/12/eabd9117 10.1126/sciadv.abd9117 PMC7968845 33731346

[11] Zhu QB, Li B, Yang DD, Liu C, Feng S (2021). A flexible ultrasensitive optoelectronic sensor array for neuromorphic vision systems. Nat. Commun..

[12] E. Chong, M. Moroni, C. Wilson, S. Shoham, S. Panzeri et al., Manipulating synthetic optogenetic odors reveals the coding logic of olfactory perception. Science **368**, 1329 (2020). https://science.sciencemag.org/content/368/6497/eaba2357 10.1126/science.aba2357 PMC8237706 32554567

[13] Yang X, Tian S, Li R, Wang W, Zhou S (2017). Use of single-crystalline Bi_2_S_3_ nanowires as room temperature ethanol sensor synthesized by hydrothermal approach. Sens. Actuators B: Chem..

[14] Fu TX (2018). Gas sensor based on three dimensional Bi_2_S_3_ nanowires network for ammonia detection at room temperature. Mater. Res. Bull..

[15] Dhar N, Syed N, Mohiuddin M, Jannat A, Zavabeti A (2018). Exfoliation behavior of van der waals strings: case study of Bi_2_S_3_. ACS Appl. Mater. Interfaces.

[16] Huang W, Xing C, Wang Y, Li Z, Wu L (2018). Facile fabrication and characterization of two-dimensional bismuth(III) sulfide nanosheets for high-performance photodetector applications under ambient conditions. Nanoscale.

[17] Han YT, Huang D, Ma YJ, He GL, Hu J (2018). Design of hetero-nanostructures on MoS_2_ nanosheets to boost NO_2_ room-temperature sensing. ACS Appl. Mater. Interfaces.

[18] Ikram M, Liu L, Liu Y, Ullah M, Ma L (2019). Controllable synthesis of MoS_2_@MoO_2_ nanonetworks for enhanced NO_2_ room temperature sensing in air. Nanoscale.

[19] Sabah FA, Ahmed NM, Hassan Z, Rasheed HS (2016). High performance CuS p-type thin film as a hydrogen gas sensor. Sens. Actuators A: Phys..

[20] Ahamad T, Naushad M, Alshheri SM (2020). Fabrication of highly porous N/S doped carbon embedded with CuO/CuS nanoparticles for NH_3_ gas sensing. Mater. Lett..

[21] Wang H, Xie KY, You Y, Hou Q, Zhang K (2019). Realizing interfacial electronic interaction within ZnS quantum dots/N-rGO heterostructures for efficient Li–CO_2_ batteries. Adv. Energy Mater..

[22] Xin X, Zhang Y, Guan X, Cao J, Li W (2019). Enhanced performances of PbS quantum-dots-modified MoS_2_ composite for NO_2_ detection at room temperature ACS Appl. Mater. Interfaces.

[23] Nguyen EP, Carey BJ, Daeneke T, Ou JZ, Latham K (2015). Investigation of two-solvent grinding-assisted liquid phase exfoliation of layered MoS_2_. Chem. Mater..

[24] Yao Y, Tolentino L, Yang Z, Song X, Zhang W (2013). High-concentration aqueous dispersions of MoS_2_. Adv. Funct. Mater..

[25] Chen XW, Wang S, Su C, Han YT, Zou C (2020). Two-dimensional Cd-doped porous Co_3_O_4_ nanosheets for enhanced room-temperature NO_2_ sensing performance. Sens. Actuators B: Chem..

[26] Han YT, Liu Y, Su C, Wang ST, Li H (2019). Interface engineered WS_2_/ZnS heterostructures for sensitive and reversible NO_2_ room temperature sensing. Sens. Actuators B: Chem..

[27] Pan Z, Cao F, Hu X, Ji X (2019). A facile method for synthesizing CuS decorated Ti_3_C_2_ MXene with enhanced performance for asymmetric supercapacitors. J. Mater. Chem. A.

[28] Clark RM, Kotsakidis JC, Weber B, Berean KJ, Carey BJ (2016). Exfoliation of quasi-stratified Bi_2_S_3_ crystals into micron-scale ultrathin corrugated nanosheets. Chem. Mater..

[29] Lu F, Li R, Yan Li N, Huo J. Yang (2015). Improving the field-effect performance of Bi_2_S_3_ single nanowires by an asymmetric device fabrication. ChemPhysChem.

[30] Ni J, Zhao Y, Liu T, Zheng H, Gao L (2014). Strongly coupled Bi_2_S_3_@CNT hybrids for robust lithium storage. Adv. Energy Mater..

[31] Hurma T, Kose S (2016). XRD Raman analysis and optical properties of CuS nanostructured film. Optik.

[32] Bera S, Katiyar AK, Sinha AK, Mondal SP, Ray SK (2016). Resistive switching characteristics of a single Zn-doped CuS nanoball anchored with multi-walled carbon nanotubes. Mater. Design.

[33] Hong C, Kim YI, Seo JH, Kim JH, Ma A (2020). Comprehensive study of the growth mechanism and photoelectrochemical activity of a BiVO_4_/Bi_2_S_3_ nanowire composite. ACS Appl. Mater. Interfaces.

[34] Liu L, Ikram M, Ma L, Zhang X, Lv H (2020). Edge-exposed MoS_2_ nanospheres assembled with SnS_2_ nanosheet to boost NO_2_ gas sensing at room temperature. J. Hazard. Mater..

[35] Meng L, He J, Tian W, Wang M, Long R (2019). Ni/Fe codoped In_2_S_3_ nanosheet arrays boost photo-electrochemical performance of planar Si photocathodes. Adv. Energy Mater..

[36] Guo Y, Yang J, Wu D, Bai H, Yang Z (2020). Au nanoparticle-embedded, nitrogen-deficient hollow mesoporous carbon nitride spheres for nitrogen photofixation. J. Mater. Chem. A.

[37] Zheng W, Xu Y, Zheng L, Yang C, Pinna N (2020). MoS_2_ van der waals p–n junctions enabling highly selective room-temperature NO_2_ sensor. Adv. Funct. Mater..

[38] Cho SY, Koh HJ, Yoo HW, Kim JS, Jung HT (2017). Tunable volatile-organic-compound sensor by using Au nanoparticle incorporation on MoS_2_. ACS Sens..

[39] Liu H, Li M, Voznyy O, Hu L, Fu Q (2014). Physically flexible, rapid-response gas sensor based on colloidal quantum dot solids. Adv. Mater..

[40] Sun Q, Wang J, Hao J, Zheng S, Wan P (2019). SnS_2_/SnS p–n heterojunctions with an accumulation layer for ultrasensitive room-temperature NO_2_ detection. Nanoscale.

[41] Cui S, Wen Z, Huang X, Chang J, Chen J (2015). Stabilizing MoS_2_ nanosheets through SnO_2_ nanocrystal decoration for high-performance gas sensing in air. Small.

[42] Qin Z, Ouyang C, Zhang J, Wan L, Wang S (2017). 2D WS_2_ nanosheets with TiO_2_ quantum dots decoration for high-performance ammonia gas sensing at room temperature. Sens. Actuators B: Chem..

[43] Chen WY, Jiang X, Lai S-N, Peroulis D, Stanciu L (2020). Nanohybrids of a MXene and transition metal dichalcogenide for selective detection of volatile organic compounds. Nat. Commun..

[44] Urs KMB, Katiyar NK, Kumar R, Biswas K, Singh AK (2020). Multi-component (Ag–Au–Cu–Pd–Pt) alloy nanoparticle-decorated p-type 2D-molybdenum disulfide (MoS_2_) for enhanced hydrogen sensing. Nanoscale.

[45] Fu D, Zhu C, Zhang X, Li C, Chen Y (2016). Two-dimensional net-like SnO_2_/ZnO heteronanostructures for high-performance H_2_S gas sensor. J. Mater. Chem. A.

[46] Hao J, Zhang D, Sun Q, Zheng S, Sun J, Wang Y (2018). Hierarchical SnS_2_/SnO_2_ nanoheterojunctions with increased active-sites and charge transfer for ultrasensitive NO_2_ detection. Nanoscale.

[47] Vaghasiya JV, Martinez CCM, Vyskočil J, Sofer Z, Pumera M (2020). Integrated Biomonitoring Sensing with Wearable Asymmetric Supercapacitors Based on Ti_3_C_2_ MXene and 1T-Phase WS_2_ Nanosheets. Adv. Funct. Mater..

