# Supplementary material for: A Novel Artificial Neuron-Like Gas Sensor Constructed from CuS Quantum Dots/Bi2S3 Nanosheets
Source: Nanomicro Lett. 2021 Dec 2;14:8. doi: 10.1007/s40820-021-00740-1 (PMC8639894; doi:10.1007/s40820-021-00740-1)
Supplement: Supplementary file 1 — Supplementary file1 (DOCX 3292 kb) [file 40820_2021_740_MOESM1_ESM.docx]

**A novel artificial neuron-like gas sensor constructed from CuS quantum dots/Bi_2_S_3_ nanosheets**

Xinwei Chen^1^, Tao Wang^1^, Jia Shi^1^, Shuyue Zheng^2^, Wen Lv^1^, Yutong Han^1^, Min Zeng^1^, Jianhua Yang^1^, Nantao Hu^1^, Yanjie Su^1^, Hao Wei^1^, Zhihua Zhou^1^, Zhi Yang^1^*, Yafei Zhang^1^*

^1^ Key Laboratory of Thin Film and Microfabrication (Ministry of Education), Department of Micro/Nano Electronics, Institute of Marine Equipment, School of Electronic Information and Electrical Engineering, Shanghai Jiao Tong University, Shanghai 200240, P. R. China.

^2^ Department of Breast Surgery, Fudan University Shanghai Cancer Center, Shanghai 200032, P. R. China.

*Corresponding authors. E-mail address: zhiyang@sjtu.edu.cn and yfzhang@sjtu.edu.cn.


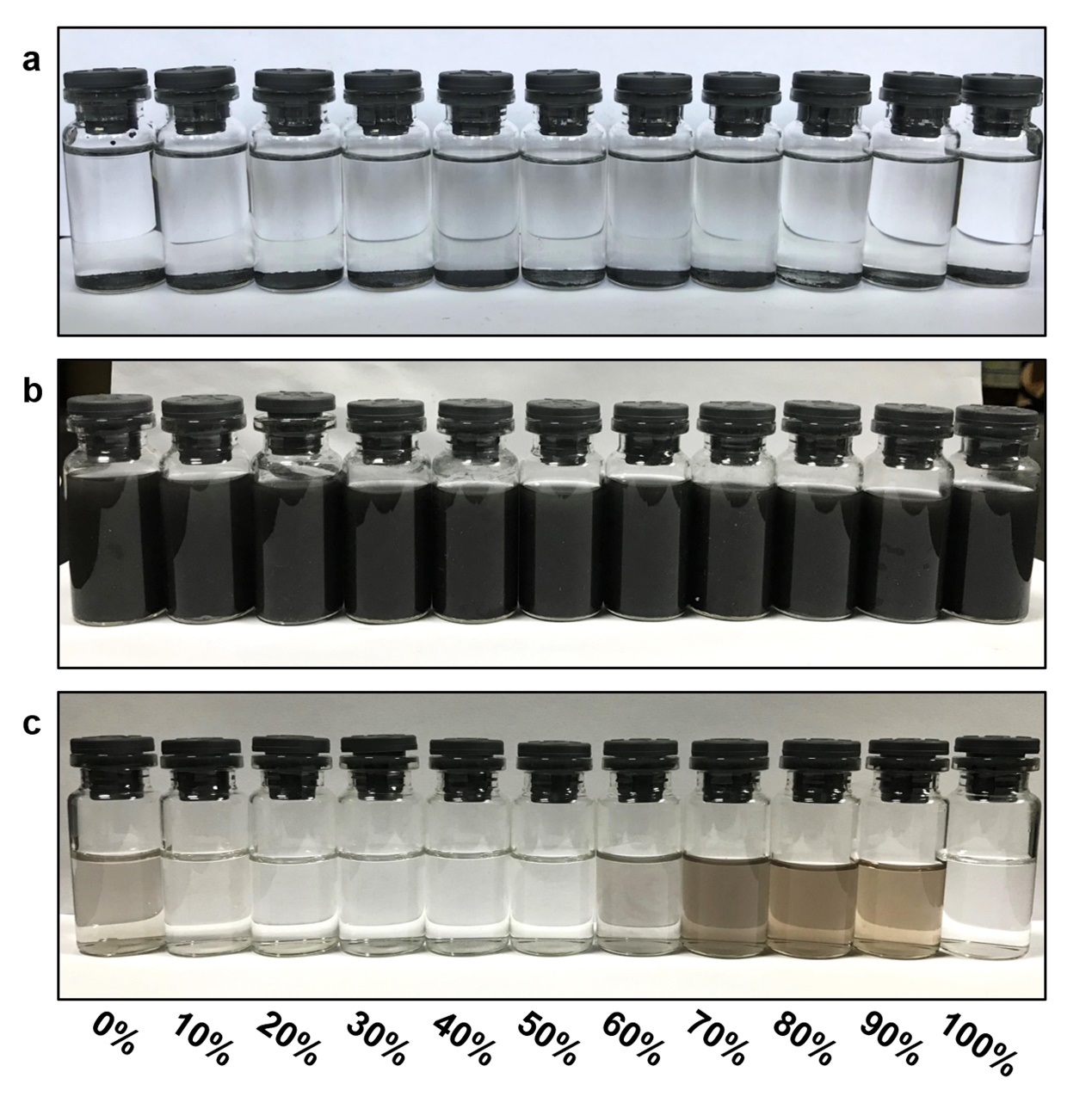


**Fig. S1** Photographs of Bi_2_S_3_ dispersions in various ethanol/water mixtures: **a** Before and **b** after ultrasonication treatment for 8 h. **c** Supernatant collection by centrifugation at 3000 rpm.


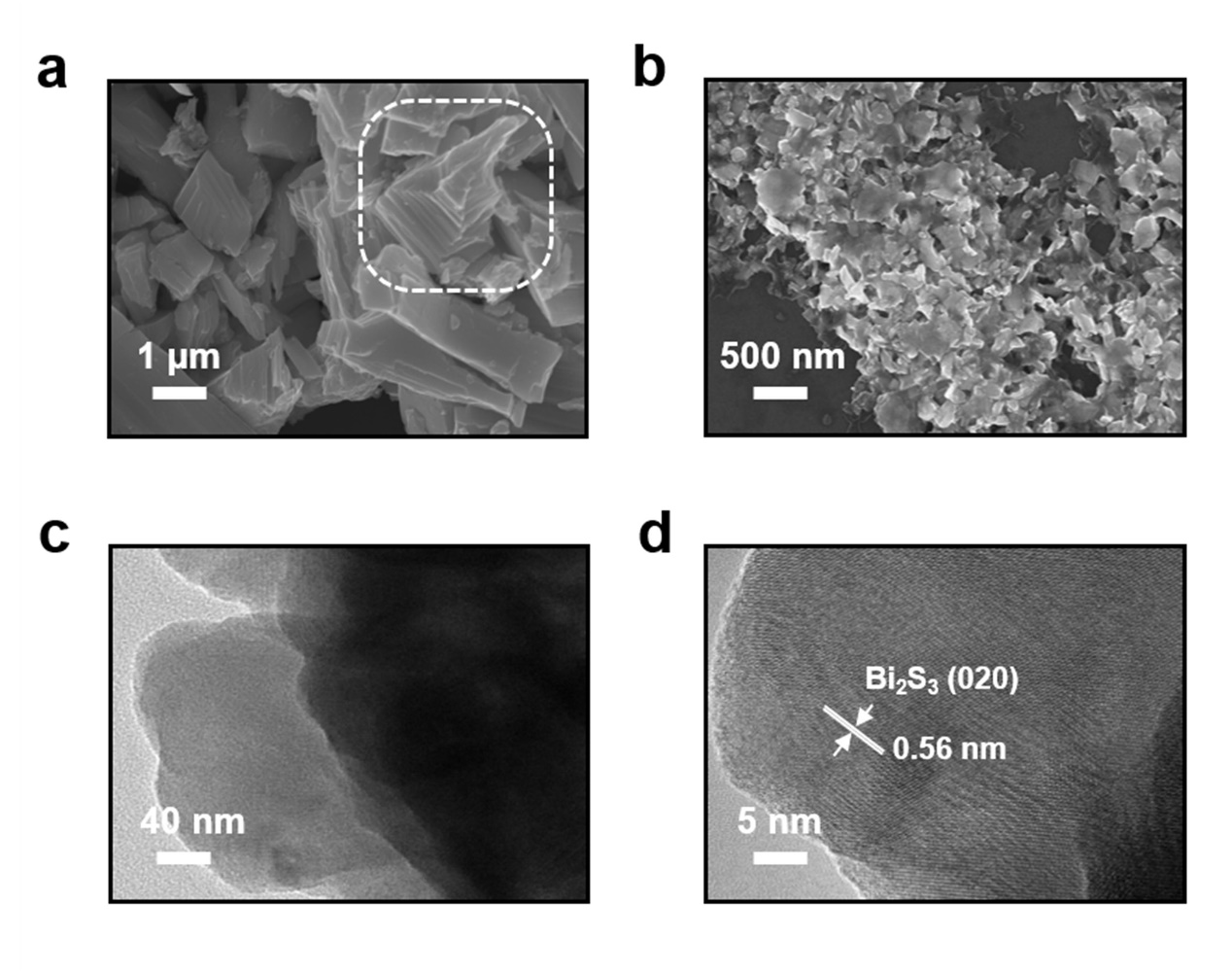


**Fig. S2** SEM image of Bi_2_S_3_ samples before **a** and after **b** Liquid phase stripping. **c** and **d** TEM and HR-TEM images of Bi_2_S_3_ NSs.


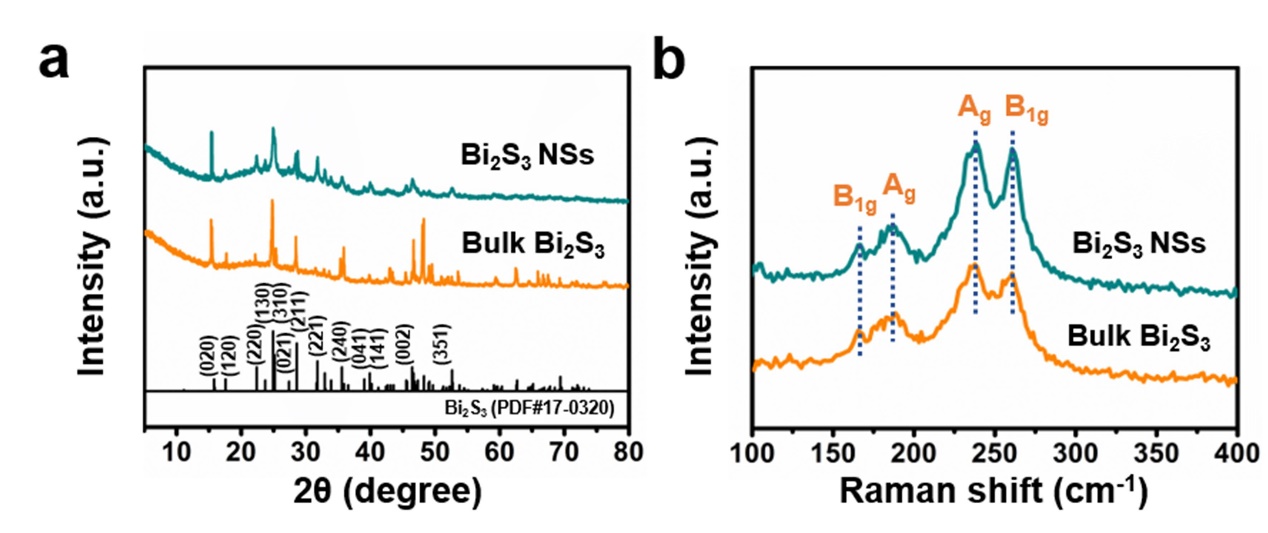


**Fig. S3** XRD patterns **a** and Raman spectra **b** of bulk Bi_2_S_3_ and Bi_2_S_3_ NSs.


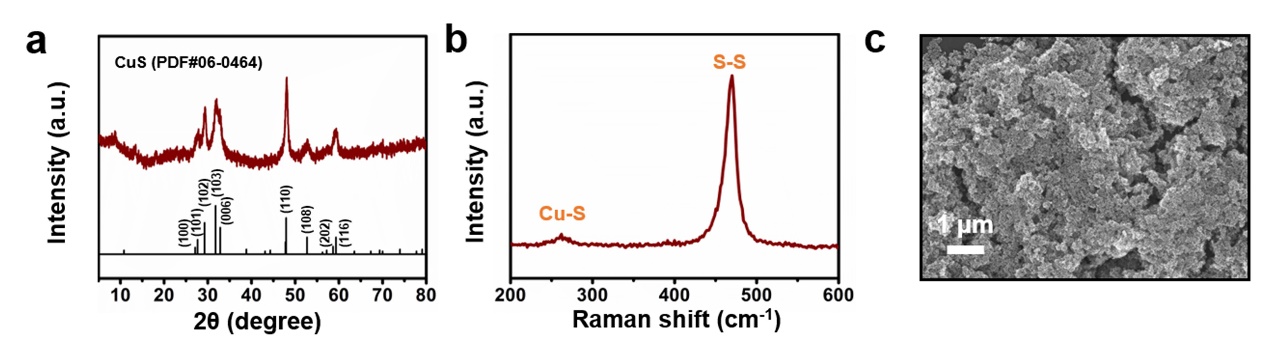


**Fig. S4 a** XRD pattern **b** Raman spectra and **c** SEM image of CuS sample.


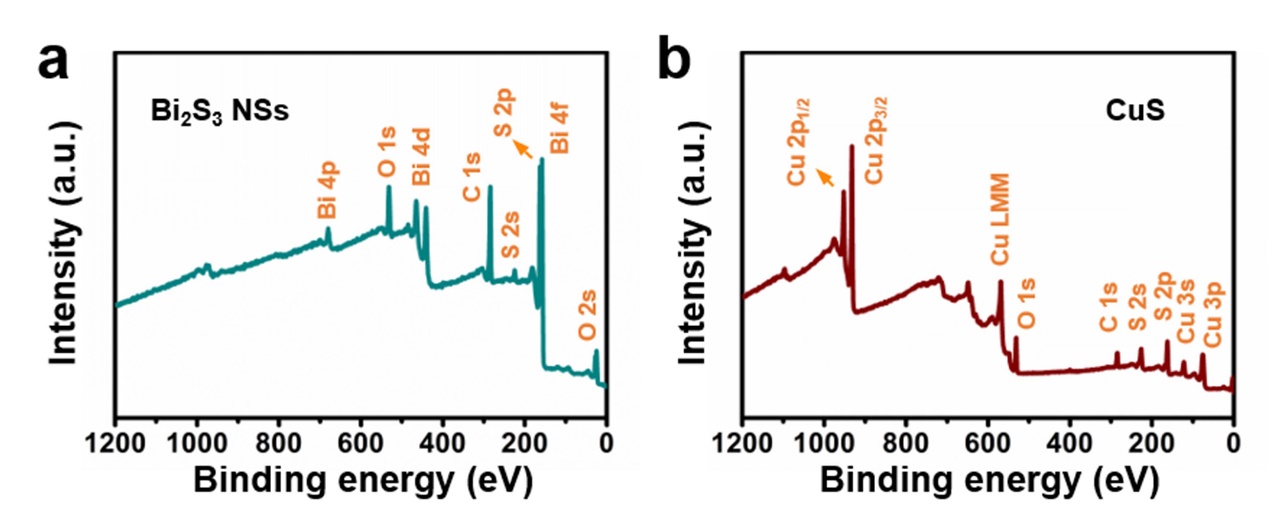


**Fig. S5** Full XPS survey spectrums of **a** Bi_2_S_3_ NSs and **b** CuS.


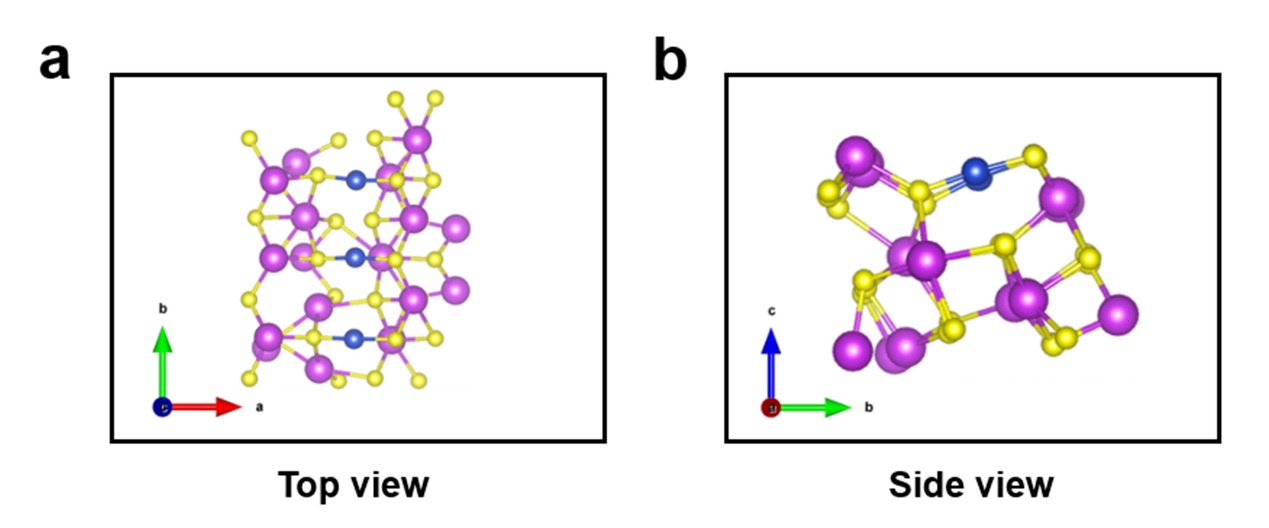


**Fig. S6** The atomic structure of CuS-Bi_2_S_3_.


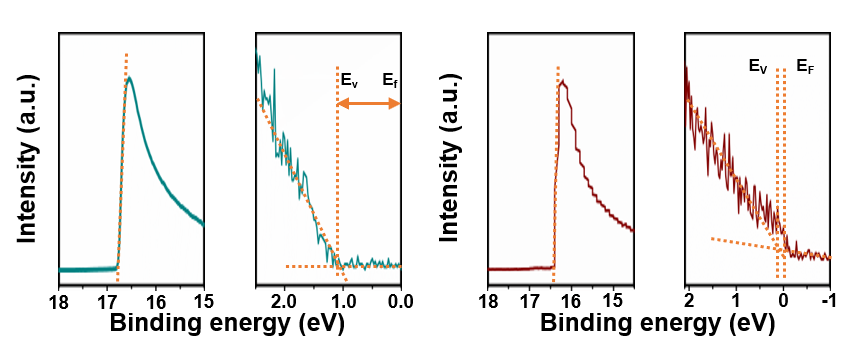


**Fig. S7** UPS spectra of **a** Bi_2_S_3_ and **b** CuS: the secondary electron cut-off energy region (left) and low binding energy region (right) of each.

**
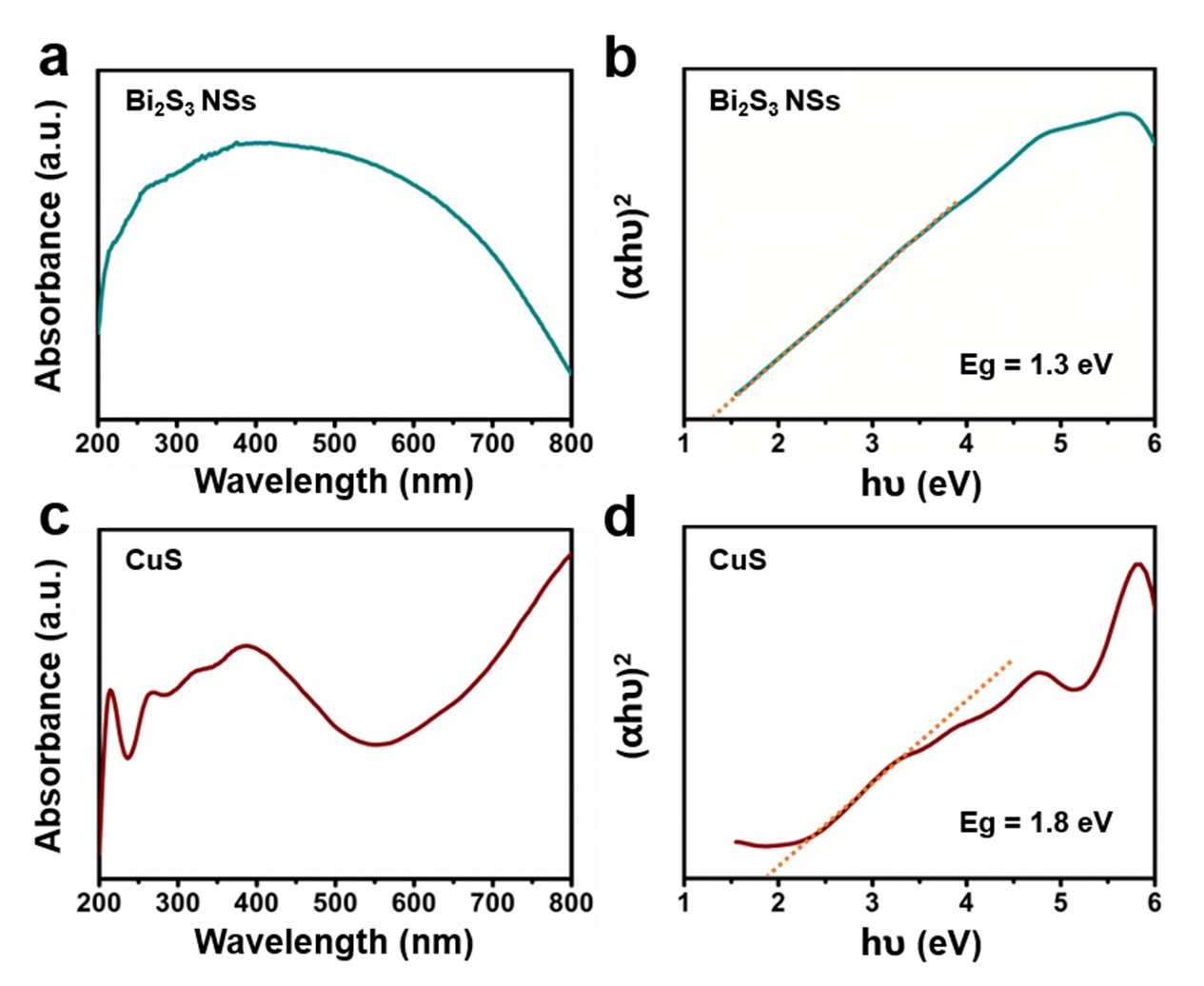
**

**Fig. S8** UV−vis diffuse reflectance spectra of **a** Bi_2_S_3_ NSs and **c** CuS. (αhν)^2^ v.s. hν curve of **b** Bi_2_S_3_ NSs and **d** CuS.

**
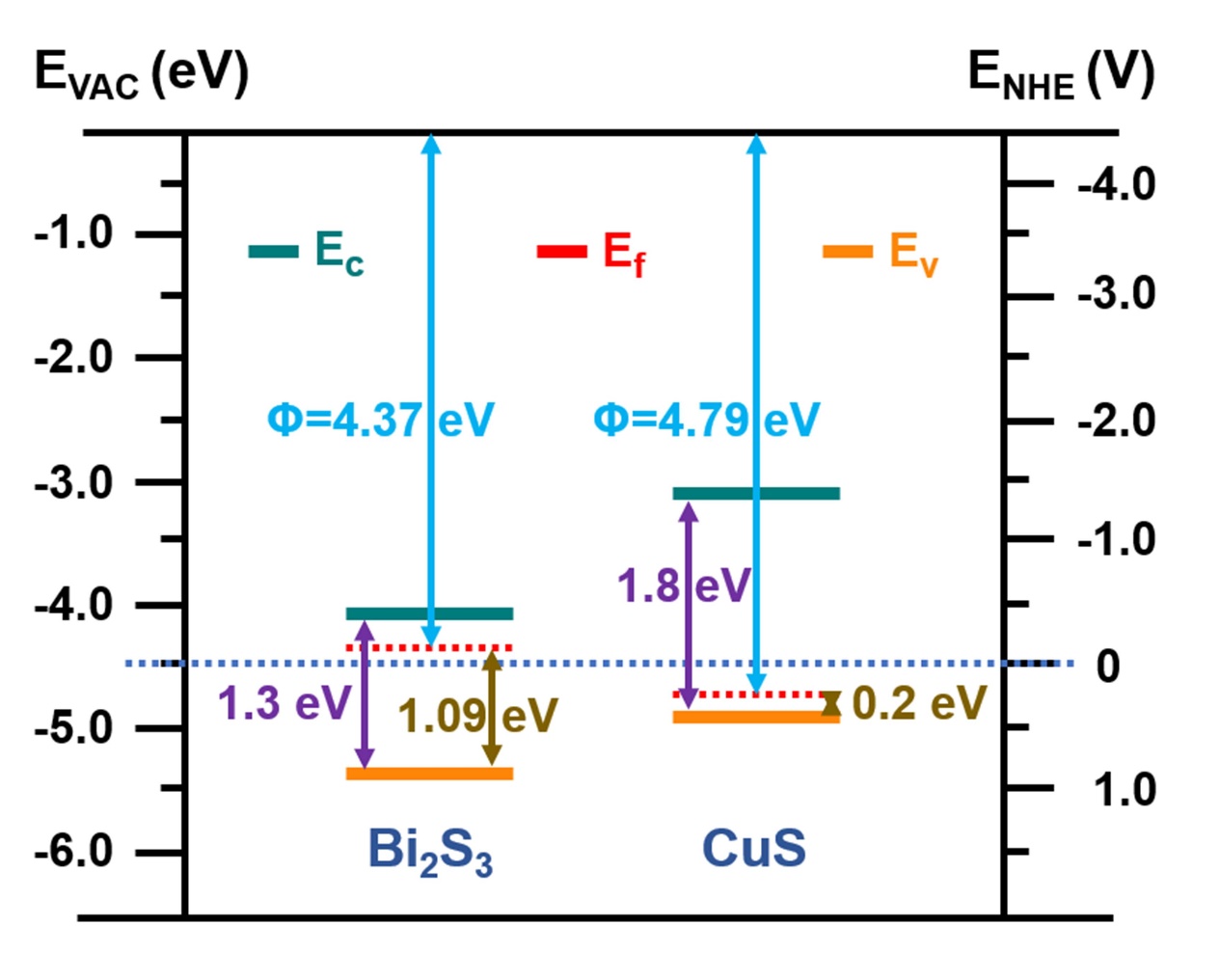
**

**Fig. S9** Energy level diagram between interfacial materials CuS and Bi_2_S_3_ and schematic diagram of the charge transfer process.


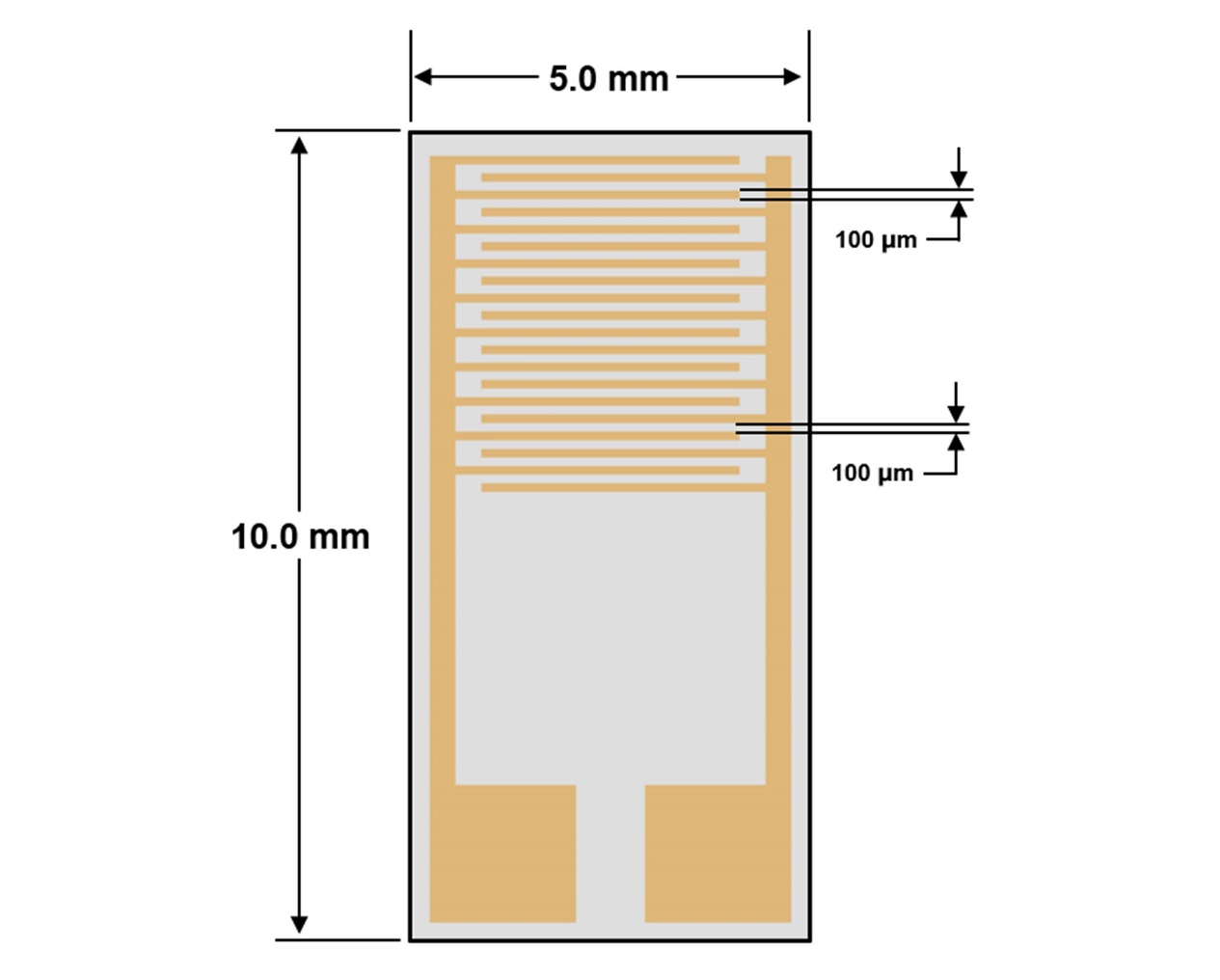


**Fig. S10** Structure diagram of the flexible interdigital electrode.

**
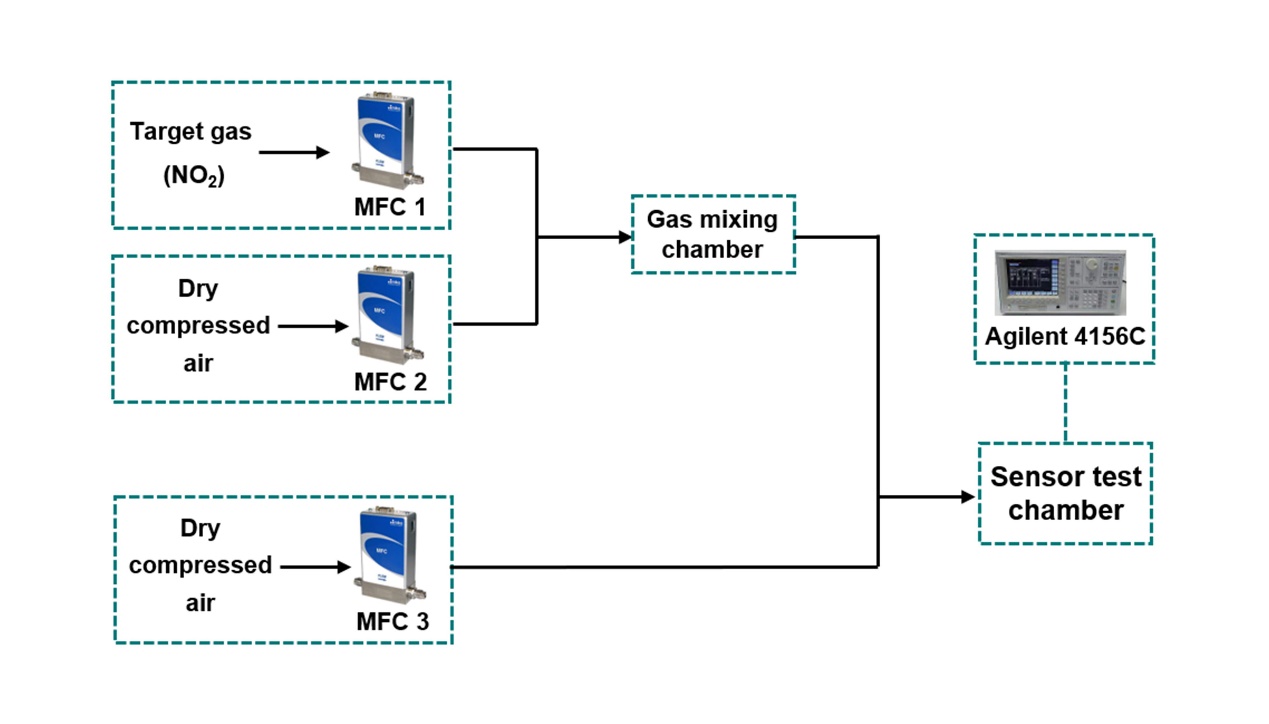
**

**Fig. S11** Schematic diagram of the homemade gas-control system.


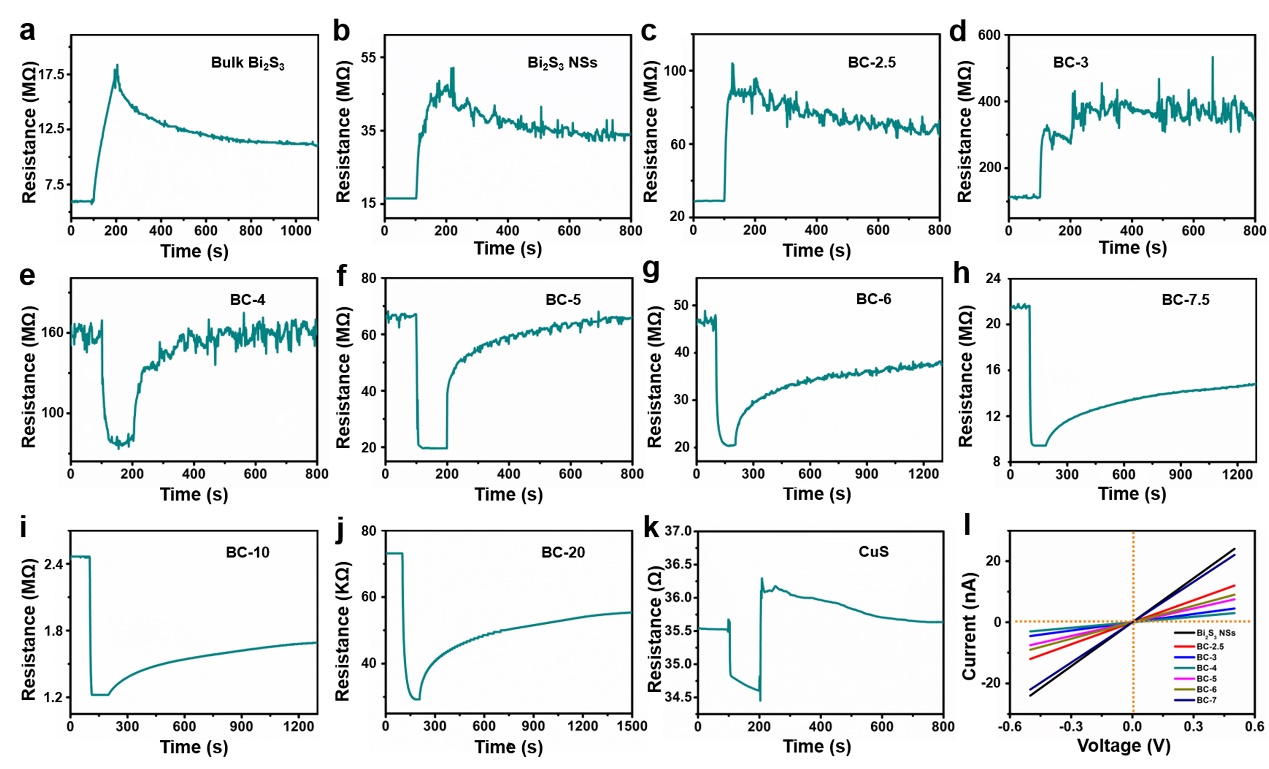


**Fig. S12 a-k** Sensitive response at a concentration to 10 ppm NO_2_ through a dynamic gas-sensing room temperature testing for bulk pure Bi_2_S_3_, Bi_2_S_3_ NSs, BC-2.5, BC-3, BC-4, BC-5, BC-6, BC-7.5, BC-10, BC-20, and pure CuS. **l** *I-V* curves of Bi_2_S_3_, different content of CuS QDs/Bi_2_S_3_ NSs and CuS-based gas sensors.

**
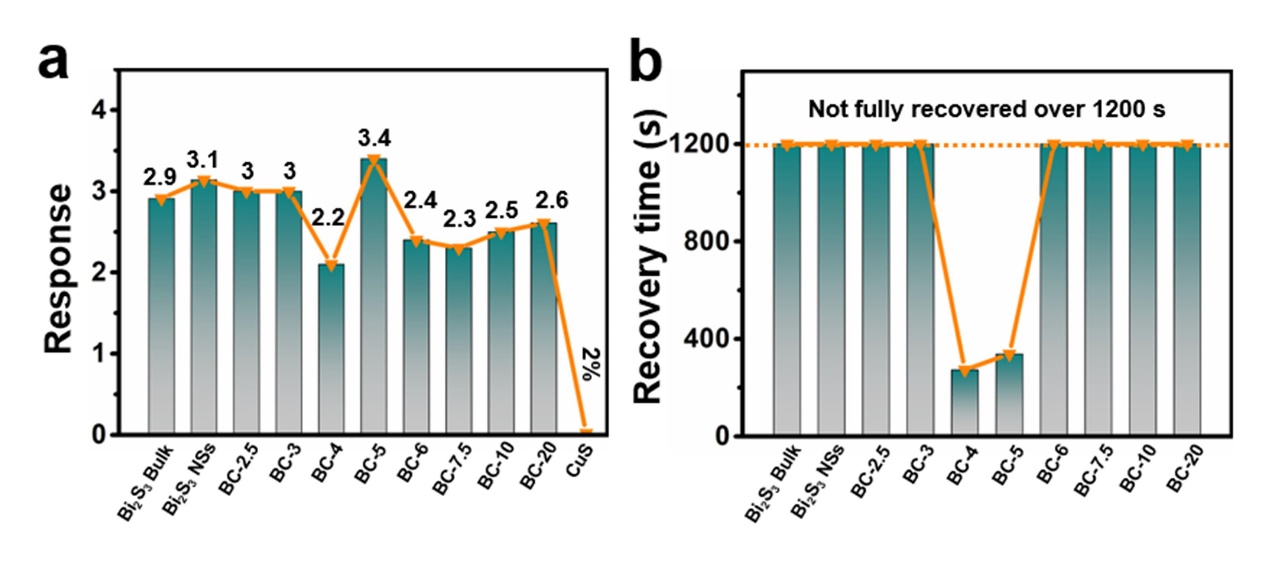
**

**Fig. S13 a** Response and **b** recovery performances of Bi_2_S_3_, different content of CuS QDs/Bi_2_S_3_ NSs, and CuS-based gas sensors to 10 ppm NO_2_.

**
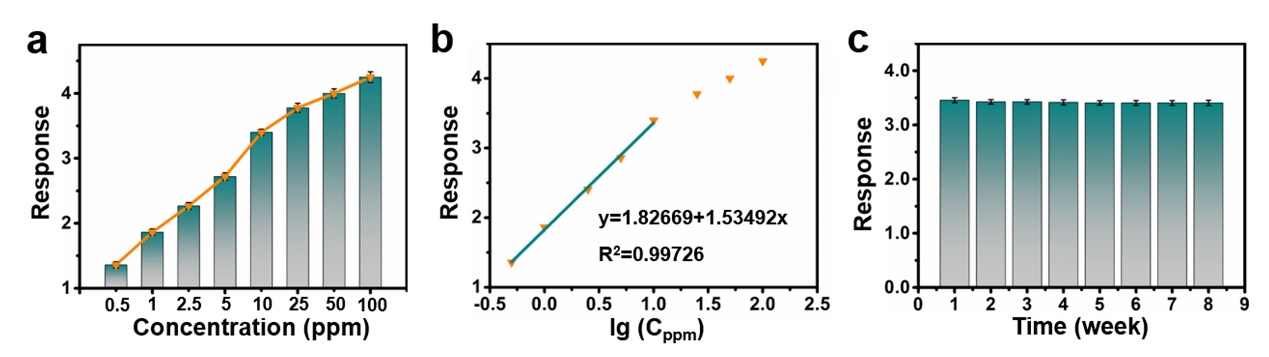
**

**Fig. S14 a** The response value of BC-5 sensor to different concentrations of NO_2_ and its error bars. **b** The response of BC-5-based sensor as a function of the logarithm of the NO_2_ concentration. **c** Long-term stability of BC-5-based sensor.


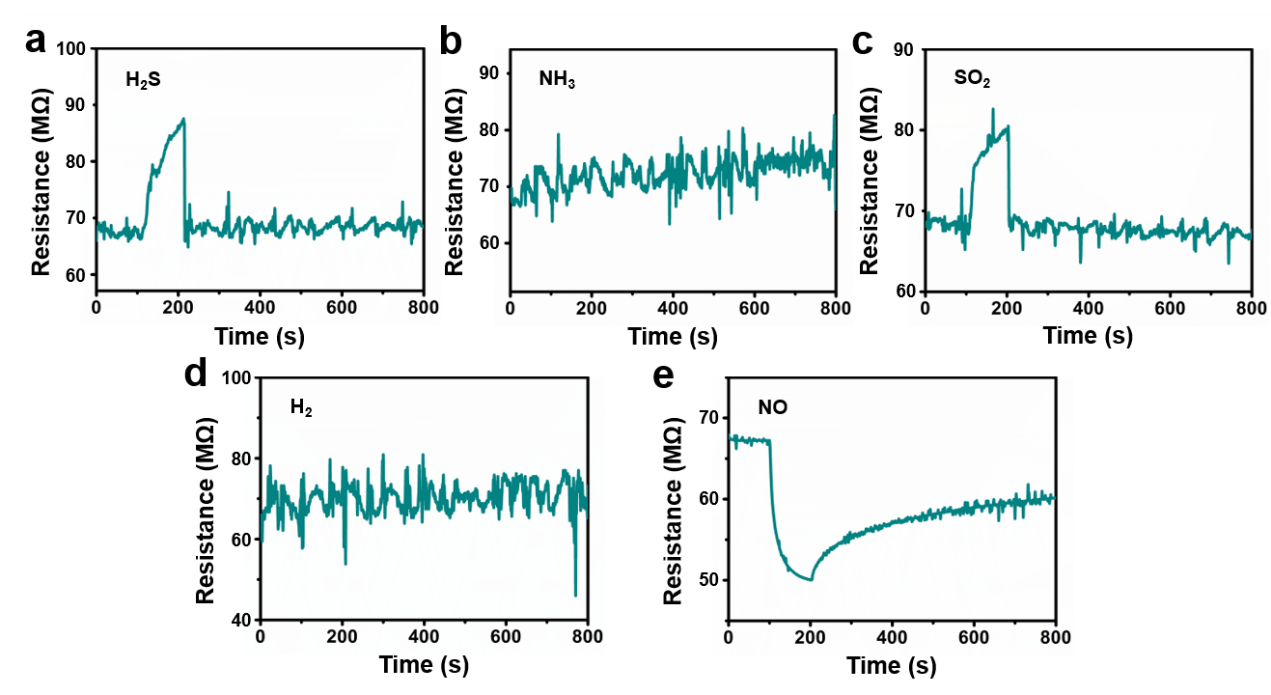


**Fig. S15** The selectivity of the BC-5-based sensor to 10 ppm different target gases of **a** H_2_S, **b** NH_3_, **c** SO_2_, **d** H_2_ and **e** NO.


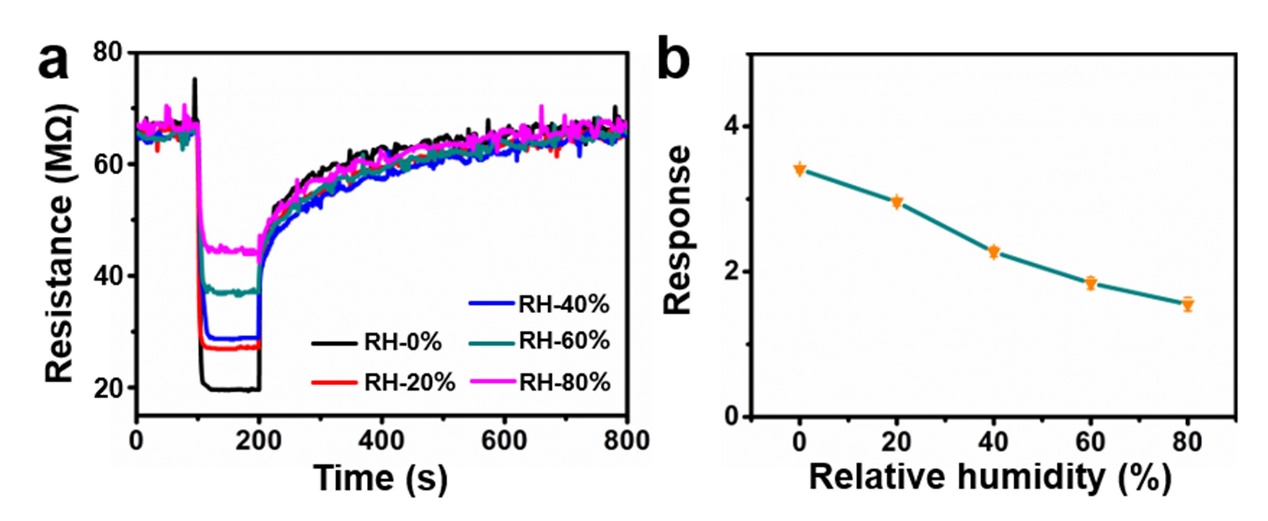


**Fig. S16** Sensing characteristics of the sensor to 10 ppm NO_2_ at different humidity levels.


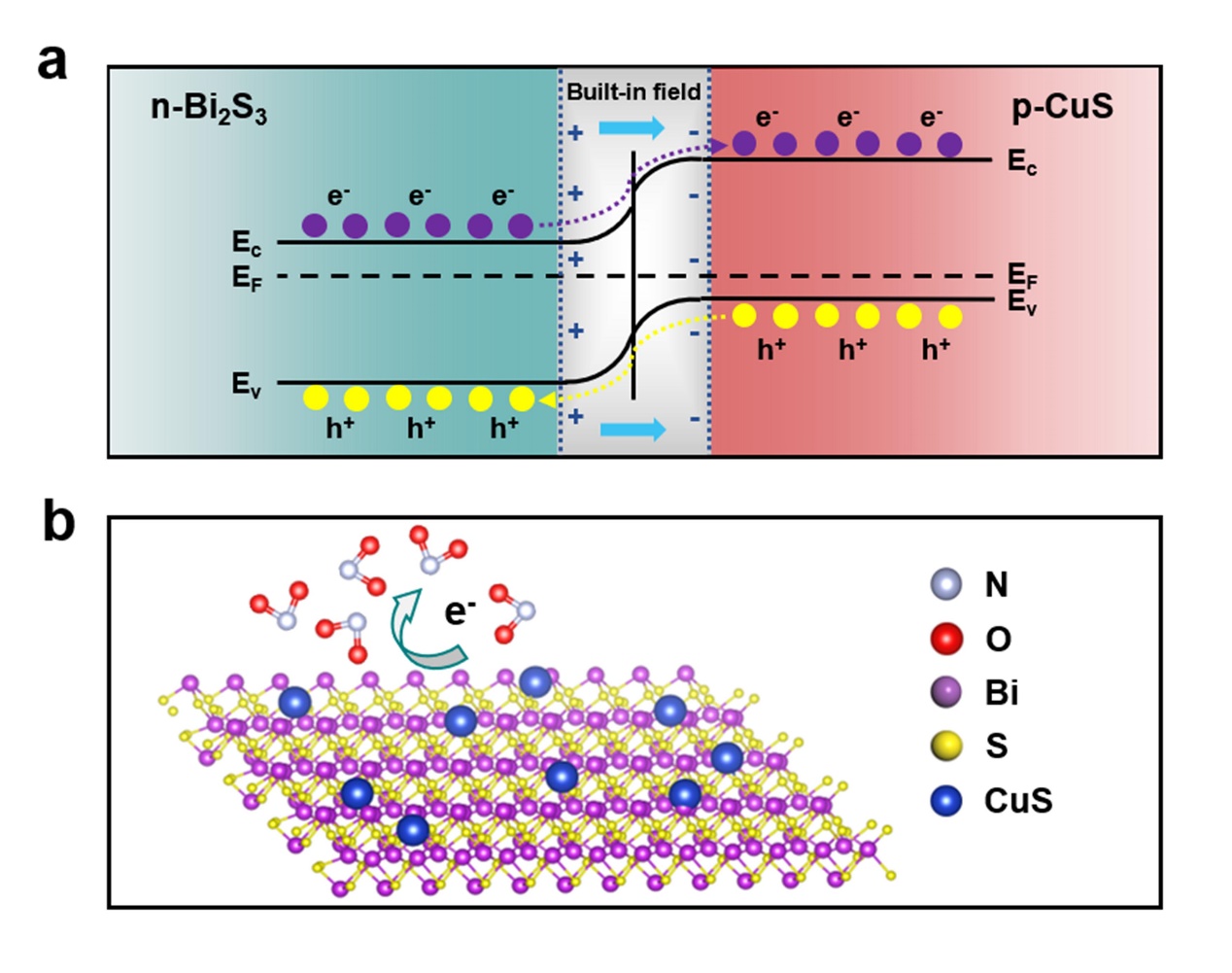


**Fig. S17** **a** Energy band structures of CuS QDs/Bi_2_S_3_ NSs heterostructure in air. **b** the proposed sensing mechanism of the heterostructure.


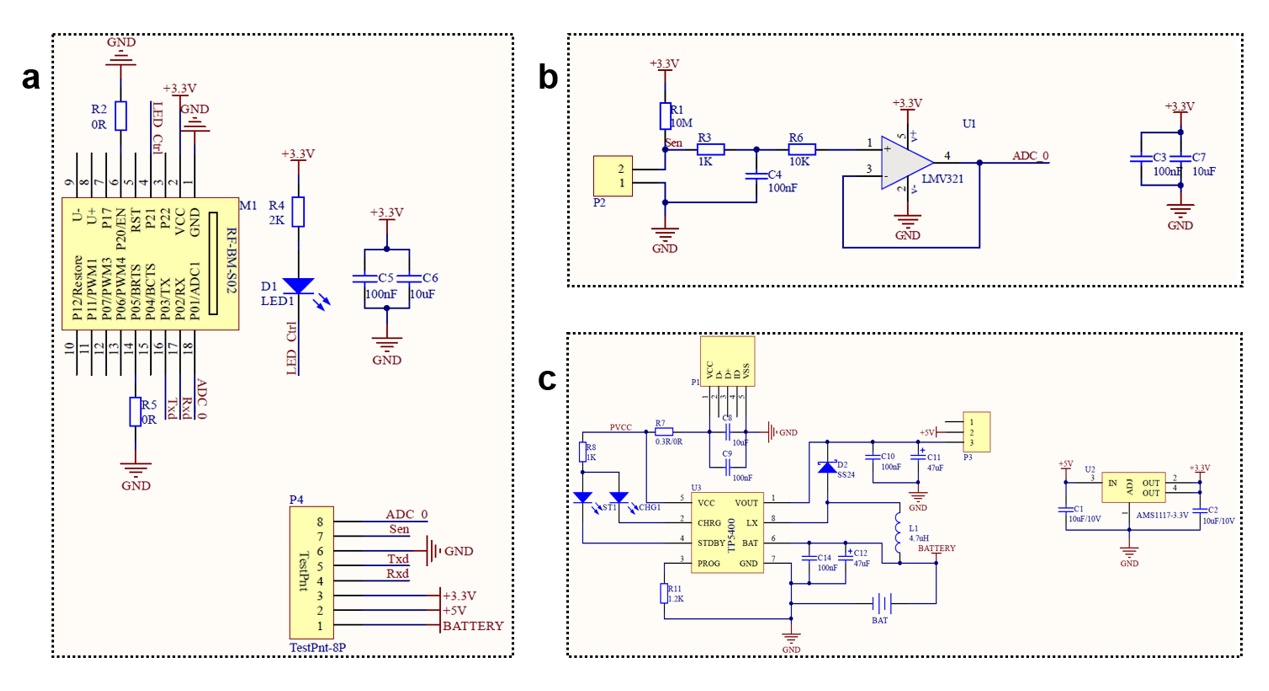


**Fig. S18** The schematic circuit diagram of the flexible printed circuit board: **a** Data acquisition and communication circuit. **b** Sensor front-end circuit. **c** USB/Wireless charging circuit.


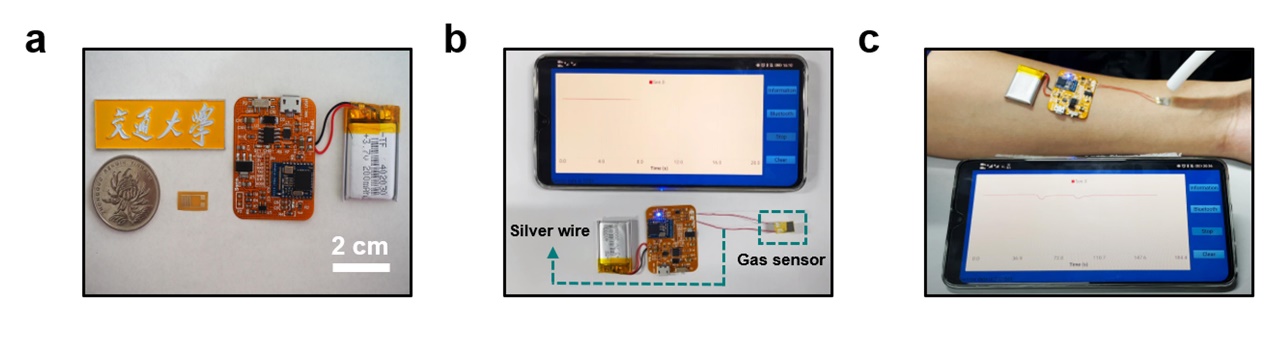


**Fig. S19** **a** Photograph of the flexible circuit and electrode used in the current work (placed next to a school logo and 1 RMB coin for comparison). **b** Photographs of Bluetooth signal connection of wearable sensor device. **c** Photographs of wireless sensor signal acquisition process of the wearable sensor device.

**Table S1** The bond length of M-O on NO_2_-CuS-Bi_2_S_3_ structure.

| Binding structure | Atoms | Bond | Length (Å) |
| --- | --- | --- | --- |
| n1 | N (from NO_2_) | Cu-N | 1.978 |
|  | Cu (CuS-Bi_2_S_3_) |  |  |
| n2 | O (from NO_2_) | Bi-O | 2.025 |
|  | Bi (CuS-Bi_2_S_3_) |  | 2.018 |
| n3 | O (from NO_2_) | Cu-O | 2.396 |
|  | Bi/Cu (CuS-Bi_2_S_3_) | Bi-O | 2.557 |
| n4 | O (from NO_2_) | Cu-O | 2.555 |
|  | Cu (CuS-Bi_2_S_3_) |  | 2.567 |

**Table S2** The charge and charge transfer of NO_2_ correlated to Fig. 5.

| Binding structure | Atoms | Charge | Charge transfer |
| --- | --- | --- | --- |
| n2 | N | 4.4197582 | -0.5802418 |
|  | O | 6.5942103 | 0.5942103 |
|  | O | 6.6273451 | 0.6273451 |
|  | Total-NO_2_ | | 0.6413136 |
| n4 | N | 4.4885298 | -0.5114702 |
|  | O | 6.598587 | 0.598587 |
|  | O | 6.6327922 | 0.6327922 |
|  | Total-NO_2_ | | 0.719909 |

**Table S3** The charge and charge transfer of CuS correlated to Fig. 3d.

| Binding structure | Atoms | Charge (e) | Charge transfer (e) |
| --- | --- | --- | --- |
| CuS-Bi_2_S_3_ | Cu1 | 10.59286 | -0.40714 |
|  | Cu2 | 10.59062 | -0.40938 |
|  | Cu3 | 10.58872 | -0.41128 |
|  | S1 | 6.800926 | 0.800926 |
|  | S2 | 6.751016 | 0.751016 |
|  | S3 | 6.768011 | 0.768011 |
|  | S4 | 6.763804 | 0.763804 |
|  | S5 | 6.771819 | 0.771819 |
|  | S6 | 6.74335 | 0.74335 |
|  | Total-CuS | | 3.371122 |

**T****able S4** Resistance values of bulk Bi_2_S_3_, Bi_2_S_3_ NSs, CuS, and different complex amounts of CuS QDs/Bi_2_S_3_ heterostructures.

|  | *R* |  |  | *R* |
| --- | --- | --- | --- | --- |
| Bulk Bi_2_S_3_ | 6.4 MΩ |  | BC-6 | 48 MΩ |
| Bi_2_S_3_ NSs | 17 MΩ |  | BC-7.5 | 21.5 MΩ |
| BC-2.5 | 30 MΩ |  | BC-10 | 2.5 MΩ |
| BC-3 | 110 MΩ |  | BC-20 | 73 KΩ |
| BC-4 | 160 MΩ |  | CuS | 35.5 Ω |
| BC-5 | 66 MΩ |  |  |  |

**Table S5** The room-temperature sensing performance comparison of NO_2_ flexible gas sensors with different sensing materials.

| Materials | Conc. (ppm) | Response | τ_res._ (s) | τ_rec._ (s) | LOD (ppb) | Ref. |
| --- | --- | --- | --- | --- | --- | --- |
| MoSe_2_ | 10 | 4 ^a^ | 250 | 150 | 10 | 1 |
| VA-2D MoS_2_ | 5 | 380%^b^ | 500 | Can’t recover | - | 2 |
| SnS_2_/S-rGO | 1 | 75% ^c^ | 600 | 1200 | 0.7 | 3 |
| RGO/Mesoporous ZnO NSs | 15 | 44% ^c^ | 140 | 630 | 43.5 | 4 |
| 3D SnS_2_-rGO | 5 | 32.1% ^d^ | 300 | Can’t recover | 2.8 | 5 |
| CuS QDs/Bi_2_S_3_ | 10 | 3.4 ^e^ | 18 | 338 | 78 | This work |

Conc.: Gas concentration; Temp.: Operating temperature; *τ_rec_*_._: Recovery time; RT: Room temperature.

^a^ *I*_g_/*I*_a_.

^b^ Δ*I*/*I*_0_

^c^ Δ*R*/*R*_a_.

^d^ Δ*G*/*G*.

^e^ *R*_g_/*R*_a_.

**References**

[1] S. Guo, D. Yang, S. Zhang, Q. Dong, B. Li, N. Tran, Z. Li, Y. Xiong, M.E. Zaghloul, Development of a cloud-based epidermal MoSe_2_ device for hazardous gas sensing. Adv. Funct. Mater. **29**, 1900138 (2019). https://doi.org/10.1002/adfm.201900138

[2] M.A. Islam, H. Li, S. Moon, S.S. Han, H.-S. Chung, J. Ma, C. Yoo, T.-J. Ko, K.H. Oh, Y.J. Jung, Y.W. Jung, Vertically aligned 2D MoS_2_ layers with strain-engineered serpentine patterns for high-performance stretchable gas sensors: experimental and theoretical demonstration. ACS Appl. Mater. Interfaces **12**(47), 53174−53183 (2020). https://doi.org/10.1021/acsami.0c17540

[3] Y. Huang, W. Jiao, Z. Chu, S. Wang, L. Chen, X. Nie, R. Wang, X. He, High sensitivity, humidity-independent, flexible NO_2_ and NH_3_ gas sensors based on SnS_2_ hybrid functional graphene ink. ACS Appl. Mater. Interfaces **12**(1), 997−1004 (2020). <https://doi.org/10.1021/acsami.9b14952>

[4] W. Li, R. Chen, W. Qi, L. Cai, Y. Sun, M. Sun, C. Li, X. Yang, L. Xiang, D. Xie, T. Ren, Reduced graphene oxide/mesoporous ZnO NSs hybrid fibers for flexible, stretchable, twisted, and wearable NO_2_ e‑textile gas sensor. ACS Sens. **4**(10), 2809−2818 (2019). https://doi.org/10.1021/acssensors.9b01509

[5] J. Wu, Z. Wu, H. Ding, Y. Wei, W. Huang, X. Yang, Z. Li, L. Qiu, X. Wang, Three-dimensional graphene hydrogel decorated with SnO_2_ for high-performance NO_2_ sensing with enhanced immunity to humidity. ACS Appl. Mater. Interfaces **12**(2), 2634−2643 (2020). https://doi.org/10.1021/acsami.9b18098
